# Distinct functional open states of the polymodal nociceptor TRPV1

**DOI:** 10.1073/pnas.2614335123

**Published:** 2026-10-15

**Authors:** Bo Hyun Lee, Shisheng Li, Simon Vu, Zhihao Yao, Yuhua Tian, Vladimir Yarov-Yarovoy, Jie Zheng

**Affiliations:** ^a^Department of Physiology and Membrane Biology, University of California Davis, School of Medicine, Davis, CA 95616; ^b^ Department of Physiology, College of Medicine, Gyeongsang National University, Jinju 52727, Republic of Korea; ^c^ Department of Pharmacology, School of Pharmacy, Qingdao University, Qingdao 266071, China; ^d^ Department of Brain Sciences, Daegu Gyeongbuk Institute of Science and Technology, Daegu 42988, Republic of Korea

**Keywords:** pain, nociception, analgesia, polymodal activation, protein conformational diversity

## Abstract

The polymodal nociceptor TRPV1 integrates diverse physical and chemical stimuli, yet whether these inputs drive a common activated state remains unresolved. Here, we show that TRPV1 activators stabilize functionally distinct open states that yield discrete single-channel conductance levels. These functional states appear to arise from distinct open-pore conformations, as evidenced by anomalous mole fraction effect measurements. Structurally, stimulus-specific changes in pore conformation were directly observed using site-directed fluorescence recordings, consistent with Rosetta-guided structural models. Notably, coactivation by two stimuli markedly slows channel opening rather than producing synergistic acceleration, suggesting potential conformational incompatibility across subunits. Together, these findings define TRPV1 gating as a stimulus-partitioned, multistate process and highlight opportunities for selective modulation of TRPV1 in pain management.

## Significance

Since the cloning of TRPV1 as a polymodal nociceptor, the prevailing view has been that diverse physical and chemical stimuli converge on a common open state. Our data, derived from multiple independent approaches under physiological conditions, challenge this assumption by revealing distinct functional open states. Direct observation of these functional states aligns with recent cryo-EM structures capturing multiple conformations compatible with ion conduction. TRPV1 is a validated, nonaddictive target for neuropathic pain; however, pharmacological efforts have been hindered by thermoregulatory side effects (hyperthermia or hypothermia) stemming from its role in sensing heat. Our findings suggest a possible path forward: selectively inhibiting open states associated with noxious stimuli while preserving heat-evoked activity, providing a conceptual framework for developing safer TRPV1-targeted analgesics.

The capsaicin receptor TRPV1 ion channel is a polymodal sensor for heat as well as toxins from poisonous plants (the vanilloids) and venomous animals (peptide toxins) (1–4), extracellular acidification associated with tissue injury and inflammation (5, 6), metal ions (7–9), and numerous synthetic compounds including 2-Aminoethoxydiphenyl borate (2-APB) (10). As a nociceptor in peripheral pain pathways and independent of the addiction-prone opioid receptor system, TRPV1 represents an attractive target for pain management. However, TRPV1 also plays a key role in thermosensation and the maintenance of core body temperature (11, 12). Consistent with this dual role, most TRPV1-targeting small-molecule drug candidates evaluated in clinical trials through systemic administration have caused severe hyperthermia (by antagonists) or hypothermia (by agonists). These outcomes highlight the need for modality-specific TRPV1-targeting therapeutics. To guide structure-based drug development targeting this attractive, nonaddictive pain sensor in a modality-specific manner, knowledge of how different stimuli activate the channel is essential.

TRPV1 is a homotetramer of stimulus-sensing subunits surrounding a central cation-permeable pore (13). Activation of TRPV1 by vanilloids is well described by Monod–Wyman–Changeux (MWC)-type allosteric gating (7, 8, 14–19). Structural studies have confirmed that pore opening occurs through a concerted transition that is progressively favored as additional resiniferatoxin (RTX) molecules bind to the channel (20). Quantitative analysis of single-channel activities under physiological conditions indicates that, during vanilloid activation, each subunit contributes approximately equally to the concerted gating transition, with an energetic contribution of about 1.8 kcal·mol^−1^ per subunit (17), whereas the vanilloid binding step itself is independent across subunits, with an intrinsic capsaicin association constant on the order of 10^6^ M^−1^ (18). The equilibrium properties of TRPV1 activation can be well-described by an MWC model using these parameters, with only a minor (<10%) correction factor to account for subunit positional effects (neighboring vs. diagonal subunits) (19). Accumulating evidence suggests that TRPV1 responds to other activating stimuli in a broadly similar allosteric framework (21). Consistent with this view, different stimuli exhibit synergistic effects on the peak activity when applied together, yet engage distinct receptive structures that are coupled to the pore through nonoverlapping allosteric pathways (3, 7, 8, 22).

Polymodal activation of TRPV1 poses a fundamental question: do diverse activation stimuli promote the same open pore state or different states? For channels activated by RTX, we have shown that there is a single conductance state regardless of the number of bound RTX molecules (17). This functional observation is consistent with cryo-EM structures resolved with different numbers of bound RTX molecules that revealed no detectable differences in the open-pore conformation (20). This behavior is expected for an MWC-type allosteric protein, in which sequential agonist binding only progressively stabilizes the relaxed (open) state without altering it (23). Nonetheless, whether different activating stimuli promote the same concerted transition to a common open state remains unresolved. This is a fundamental mechanistic question for polymodal receptors; it is particularly important for TRPV1 given that both the selectivity filter and the S6 bundle crossing of TRPV1 are thought to function as activation gates (24–27). Although evidence for the existence of distinct open states has begun to emerge (28), no systematic investigation into this fundamental question has been reported.

Here, we combined multiple experimental approaches to investigate the ion permeation properties of open TRPV1 channels activated by diverse stimuli, the associated conformational changes and the activation kinetics. Data from these experiments reveal clearly distinct open-pore states that exhibit apparent conformational clashes. These findings point to potential strategies for selective channel inhibition for clinical purposes.

## Results

### TRPV1 Activators Induce Distinct Ion Conductance States.

**Fig. 1.**
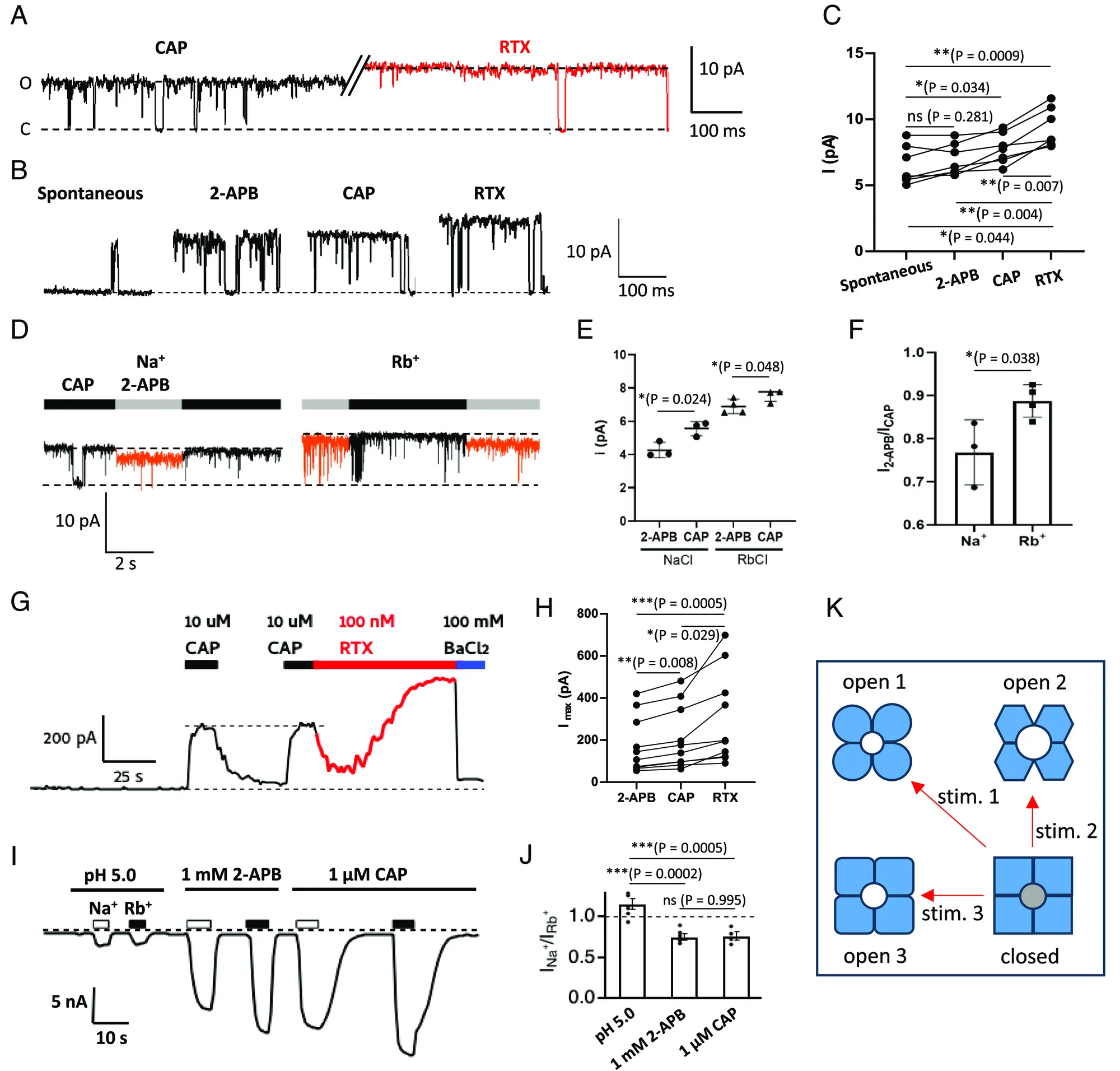
TRPV1 exhibits different conducting states when activated by different agonists. (*A*) Comparison between the single-channel amplitudes from a channel activated by capsaicin (CAP, 10 μM) and RTX (100 nM) within the same inside-out patch-clamp recording of mouse TRPV1. (*B* and *C*) Comparison between the single-channel amplitudes of spontaneous events and events activated by 2-APB (3 mM), capsaicin (10 μM), and RTX (100 nM) within the same inside-out patch-clamp recording of mouse TRPV1. n = 7. (*D*–*F*) Comparison between the single-channel amplitudes activated by capsaicin (10 μM) and 2-APB (3 mM) with different permeate ions (Na^+^ or Rb^+^). n = 3 to 4. (*G*) Representative inside-out macroscopic current of mouse TRPV1 activated by saturating concentrations of capsaicin (10 μM) and RTX (100 nM). (*H*) Summary of the amplitudes of macroscopic current activated by saturating concentrations of 2-APB, capsaicin, and RTX. n = 9. (*I* and *J*) Representative whole-cell patch-clamp current trace and comparison of amplitude ratio between current carried by Na^+^ and Rb^+^ ions induced by different activators: protons (pH 5.0), 2-APB (1 mM), and capsaicin (1 μM). n = 4 to 6. (*K*) Schematic illustration of the model for channel activation by different stimuli. All currents were recorded at +80 mV, except for those in panel *I*, which were recorded at −80 mV. Data are presented as mean ± SEM. Statistical significance was determined by repeated-measures (panels *C* and *H*) or ordinary (panel *J*) one-way ANOVA followed by Tukey’s multiple-comparisons test, or paired Student’s *t* test (panel *E*). *P < 0.05; **P < 0.01; ***P < 0.001; ns, not significant.

To functionally assess the activated state of TRPV1, we first examined single-channel conductance in response to diverse chemical stimuli. An intriguing observation was that, in the same continuous recording, the single-channel amplitude increased substantially when the perfusate contained RTX instead of capsaicin (Fig. 1*A*). Close comparisons further revealed that ligand-induced openings in general had a higher amplitude than spontaneous opening events, and different ligands yielded different open-event amplitudes (Fig. 1 *B* and *C* and *SI Appendix*, Fig. S1*A*). This phenomenon could be readily reproduced with different permeant ions (Fig. 1 *D* and *E* and *SI Appendix*, Fig. S1*B*), with the amplitude ratio being permeant ion dependent (Fig. 1*F*). Since varying ligand concentrations did not alter the amplitude of single-channel opening events, ligand-dependent current amplitudes could not be due to pore block by the activators. To assess whether the single-channel amplitude could be affected by a filtering effect on short opening events (29), open channel noise was measured in the presence of different activators and compared (*SI Appendix*, Fig. S2 *A* and *B*). We found that, while open-channel events induced by 2-APB appeared to exhibit excess noise, capsaicin and RTX induced open events had the same noise level as spontaneous open events. All three ligands could open the channel to near unity probabilities (*SI Appendix*, Fig. S2*C*). A difference in amplitude could not be confidently detected for open events induced by extracellular protons (at pH6.5) alone and those induced by capsaicin (1 μM) and protons together (*SI Appendix*, Fig. S3). TRPV1 activated by heat exhibited a higher conductance level, which we have previously shown to be temperature-dependent (30).

We recently discovered that a Y511A mutation makes RTX binding reversible, hence concatemers containing varying combinations of wild type (WT) and Y511A mutant subunits (31) offer a means to lock a fixed number of RTX molecules in a channel for equilibrium measurements (17, 18). Using this approach, we found that there was no difference in single-channel amplitude when the open channel contained one, two, three, or four RTX molecules (*SI Appendix*, Fig. S4). To further confirm that single-channel amplitude does not change by sequential ligand binding, we monitored current amplitude during the slow (seconds to minutes) RTX binding process (*SI Appendix*, Fig. S5*A*). As we have previously observed from the WT channels (17), current amplitude remained the same when RTX bound progressively to concatemer channels (*SI Appendix*, Fig. S5*B*). Therefore, conductance differences were due to the type of ligands, not the number of bound ligands.

Distinct single-channel amplitudes suggest that the macroscopic current amplitude would also vary with activators when the open probability reaches the maximal level. We found that this was indeed the case. Recorded from the same membrane patch in the presence of ligands at saturating concentrations that produced near unity open probabilities (*SI Appendix*, Fig. S2*C*), the stable peak current amplitude induced by RTX was substantially higher than that induced by capsaicin (Fig. 1 *G* and *H*). Similar observations were made using 2-APB and protons (Fig. 1 *I* and *J*). We conclude that, when used individually, different ligands promote TRPV1 into different activated states that exhibit distinct conductance properties (Fig. 1*K*).

### Anomalous Mole Fraction Effect (AMFE) Revealed Distinct Pore Conformations.

**Fig. 2.**
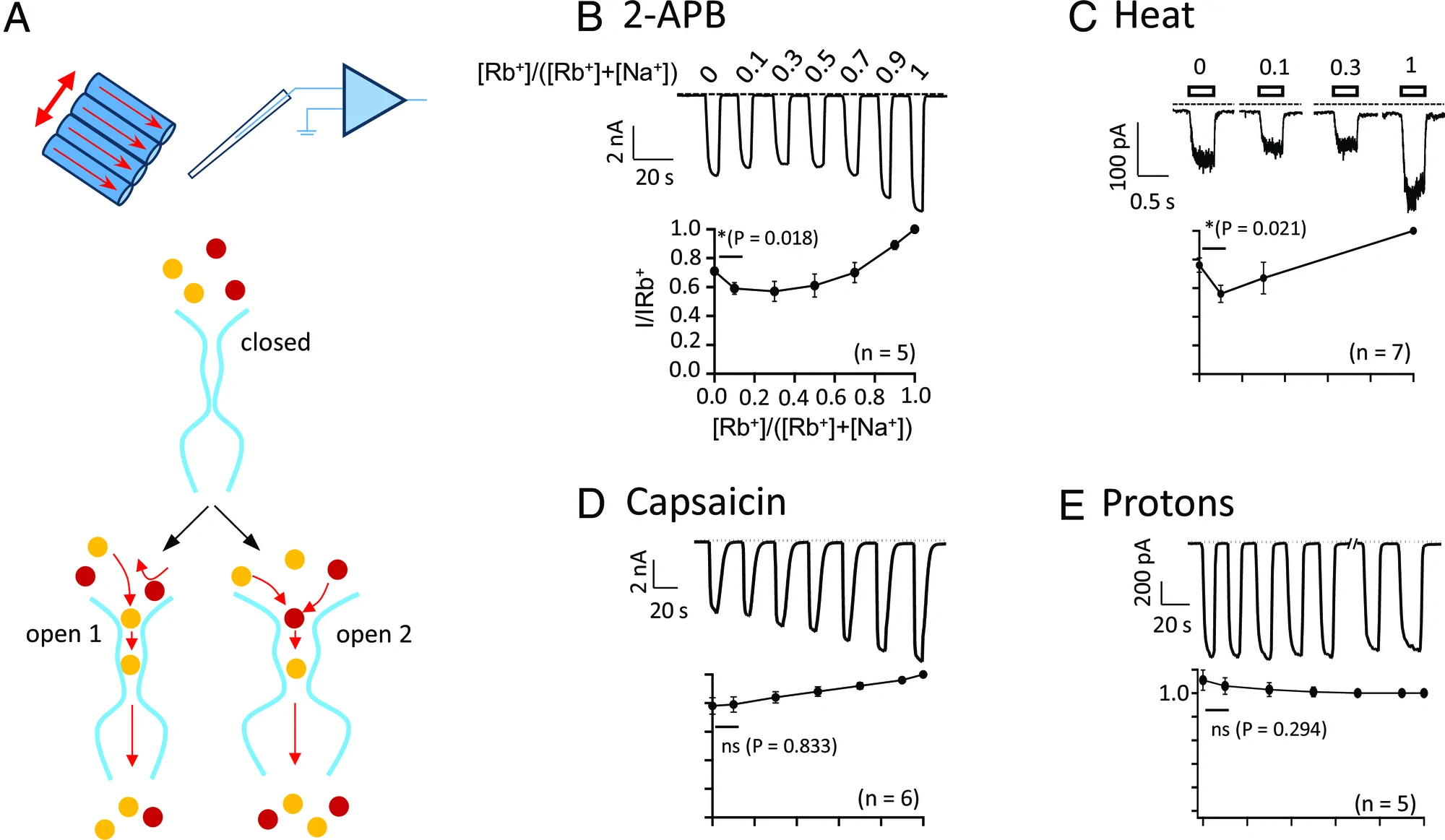
AMFEs in TRPV1 depend on the types of activators used. (*A*) *Top*, diagram of patch-clamp recording setup; seven solutions with varying Na^+^/Rb^+^ ratios were prepared and applied to the cell via parallel glass barrels. *Bottom*, two models of the channel pore showing ion–ion interference, one with single-ion occupancy (open 1) and the other with multi-ion occupancy (open 2). (*B*–*E*) *Top*, representative current traces induced by 1 mM 2-APB (*B*), heat (*C*), 1 μM capsaicin (*D*), or pH 5.0 (*E*) at varying Na^+^/Rb^+^ ratios ([Na^+^] + [Rb^+^] = 140 mM). *Bottom*, the corresponding current–ratio relationship. All data are presented as mean ± SEM. Statistical significance was determined using Student’s *t* test. *P < 0.05; ns, not significant.

One of the most sensitive functional tests of pore conformation is the AMFE (32). The approach uses macroscopic current recording to monitor ion–ion interaction within the pore as they traverse it—when two permeant ion species are present, interference between them will mutually slow down the passage of each, leading to a reduction in current amplitude and a U-shaped current amplitude plot upon permeant ions titration. Hence AMFE is expected to be sensitive to subtle differences in the pore structure that affect ion–ion or ion–pore interactions (Fig. 2*A*). To detect the AMFE, we measured current amplitude in a series of solutions containing two permeant ions, Na^+^ and Rb^+^, at varying ratios but the same total concentration (140 mM). We observed clear AMFE in TRPV1 using 2-APB: The inward current amplitude at a constant 2-APB concentration (1 mM) exhibited a U-shaped dependence on ion concentration ratio, characteristic of AMFE (Fig. 2*B*). Similarly, heat-activated currents exhibited a U-shaped ion concentration ratio dependence (Fig. 2*C*).

However, the U-shaped relationship was absent when capsaicin (Fig. 2*D*) or protons (Fig. 2*E*) were used as the activator. Using the same experimental conditions as described above, capsaicin- and proton-induced currents showed monotonic changes in amplitude as the Na^+^:Rb^+^ molar ratio increased—rising for capsaicin but falling for protons. The absence of AMFE was not due to a difference in channel open probability because a fixed agonist concentration was used in each recording. Furthermore, no AMFE could be produced when we increased or decreased the agonist concentration. Similar observations were made by replacing capsaicin with RTX and altering the orders of solution applications (*SI Appendix*, Fig. S6). AMFE measurements confirmed that the open conformations adopted by the TRPV1 pore depend on the activators used and differ sufficiently for permeant ions to sense these differences as they pass through.

### Site-Specific Fluorescence Recording Reveals Distinct Activation Conformational Changes.

**Fig. 3.**
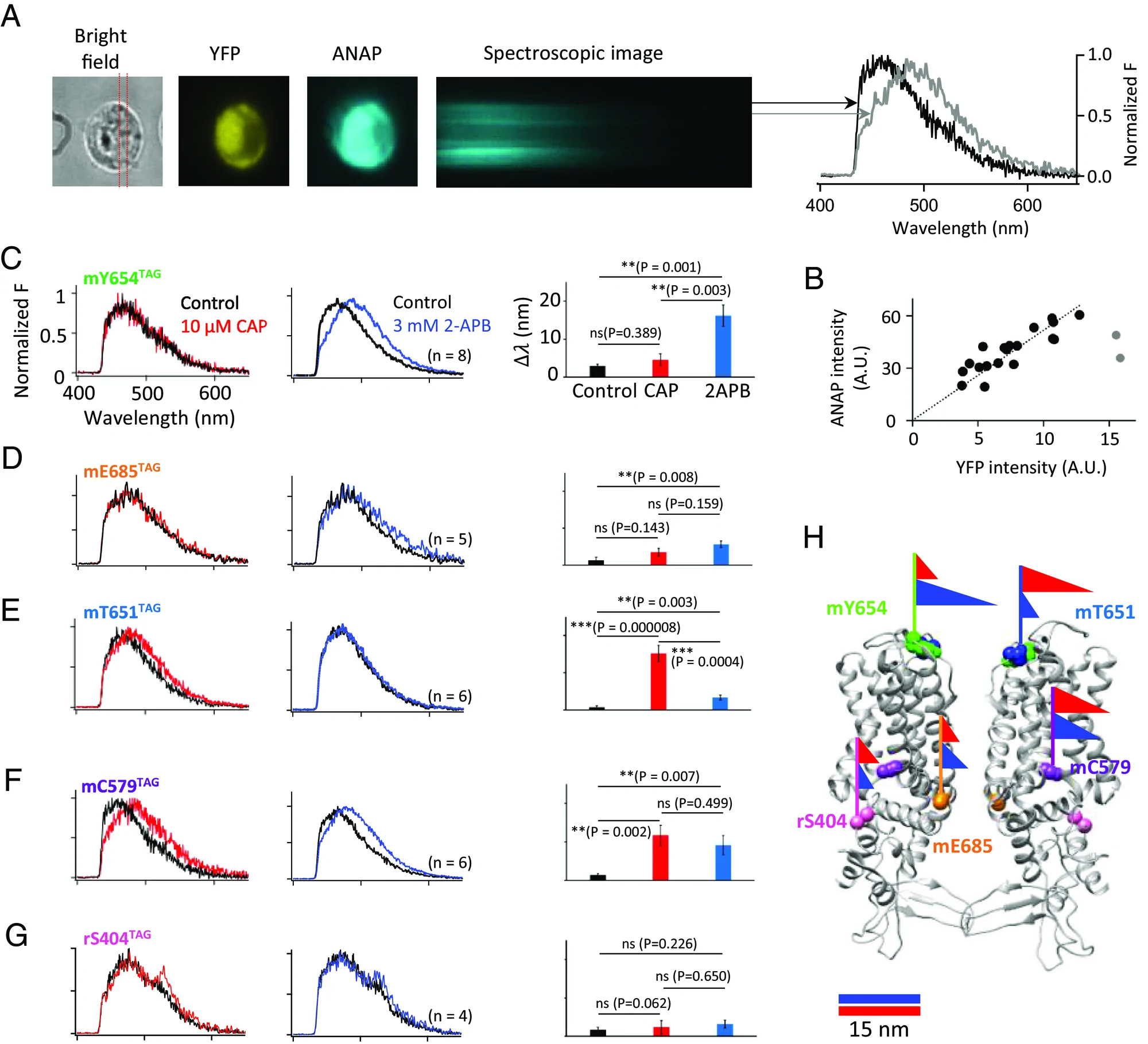
Site-specific fluorescence measurements during TRPV1 activation reveals different conformational changes in the outer pore induced by capsaicin and 2-APB. (*A*) Bright-field (1st panel) and fluorescence images (2nd and 3rd panels) of a representative HEK293T cell expressing C579^TAG^ TRPV1 incorporated ANAP. The red dashed box in the 1st panel indicates the position of the spectrograph input slit from which fluorescence emission was collected. *Right* panel, fluorescence spectral image of the ANAP emission. Black arrow indicates the position of the cell plasma membrane where the fluorescence emission spectrum was measured. (*B*) Correlation between ANAP fluorescence and YFP fluorescence. (*C*–*G*) Representative ANAP emission spectra of Y654^TAG^ (*C*), E685^TAG^ (*D*), T651^TAG^ (*E*), C579^TAG^ (*F*), and S404^TAG^ (*E*), measured in the presence of 10 μM capsaicin (red, *Left* panel) or 3 mM 2-APB (blue, *Middle* panel). *Right* panel, Comparison of shifts in the emission peak position under different conditions. (*H*) Mapping of conformational changes indicated by spectral shifts onto the TRPV1 cryo-EM structure (3J5P). The length of flags represents the magnitude of shift; red and blue flags represent capsaicin and 2-APB induced movements, respectively. Error bars indicate mean ± SEM. Statistical significance in *C*–*G* was determined using Student’s *t* test. **P < 0.01 and ***P < 0.001; ns, not significant.

The observations described above suggest that various activators would induce distinct activation conformational changes. To directly detect dynamic conformational changes at selected locations within the channel under physiological conditions, we set up site-specific fluorescence measurements. Nonnatural amino acid carrying an ANAP fluorophore was introduced into TRPV1 at selected sites using the amber stop codon strategy (33, 34). We screened 28 candidate positions and identified 8 positions at which introducing an ANAP-carrying nonnatural amino acid did not disrupt channel expression or function (*SI Appendix*, Table S1), as judged by extensive fluorescence and current characterizations (*SI Appendix*, Figs. S7–S10). Faithful incorporation of a nonnatural amino acid was confirmed by the characteristic ANAP emission spectrum at the plasma membrane (Fig. 3*A*) and the correlation between ANAP fluorescence intensity and that of a YFP tag on the channel (Fig. 3*B*). ANAP fluorescence signals were selectively collected from properly assembled and functional channels in the plasma membrane region with a spectrograph (35) (Fig. 3*A*), which were distinct in spectral properties from that of free ANAP inside the cell (*SI Appendix*, Fig. S8 and Table S2). The signature spectrum of ANAP further served as a sensitive reporter of the local environment as the channel underwent conformational changes (Fig. 3 *C*–*G* and *SI Appendix*, Fig. S10).

As expected, ANAP fluorescence changed upon channel activation gating. The fluorescence spectrum of ANAP attached to two sites along the pore—Y654 at the outer pore region, and E685 at the inner pore region—exhibited a clear right shift when 2-APB was applied to activate the channels (Fig. 3 *C* and *D*). ANAP emission is highly sensitive to the local environment of the fluorophore; a rightward spectral shift occurs when local hydrophilicity increases, which is expected when an ANAP-labeled residue becomes more solvent-exposed in the open conformation (36). We previously examined these sites following capsaicin application (26). Interestingly, the spectral shift induced by capsaicin was clearly less prominent at Y654, but not statistically distinguishable at E685 (Fig. 3 *C* and *D*), suggesting that pore opening induced by 2-APB and capsaicin is clearly distinct at the outer pore but shows much less divergence at the inner pore. This observation is particularly interesting because the outer pore movement is an activation step closely tied to the opening of the selectivity filter gate (20, 24, 26, 37), a structure that directly affects ion conductance.

To further confirm the outer pore conformational changes induced by capsaicin and 2-APB were indeed different, we compared fluorescence measurements at another outer pore site, T651, where ANAP-carrying nonnatural amino acid incorporation yielded functional channels. A difference was again observed but in the opposite direction, with the capsaicin-induced change being more prominent compared to the 2-APB-induced change (Fig. 3*E*). The difference in conformational change at these outer pore sites appeared to be authentic: At C579, which is near both the binding site for capsaicin and the anticipated 2-APB binding site (based on cryo-EM data for TRPV2 and TRPV3), the fluorescence changes upon channel activation were substantial but similar in magnitude (Fig. 3*F*), whereas at the channel peripheral site S404, no major change was observed with either stimulus (Fig. 3*G*). Based on these observations, the stimulus-dependent fluorescence signals likely arose from distinct conformational changes induced by different stimuli (Fig. 3*H*).

### Computational Modeling of the Open States Induced by Different Ligands.

**Fig. 4.**
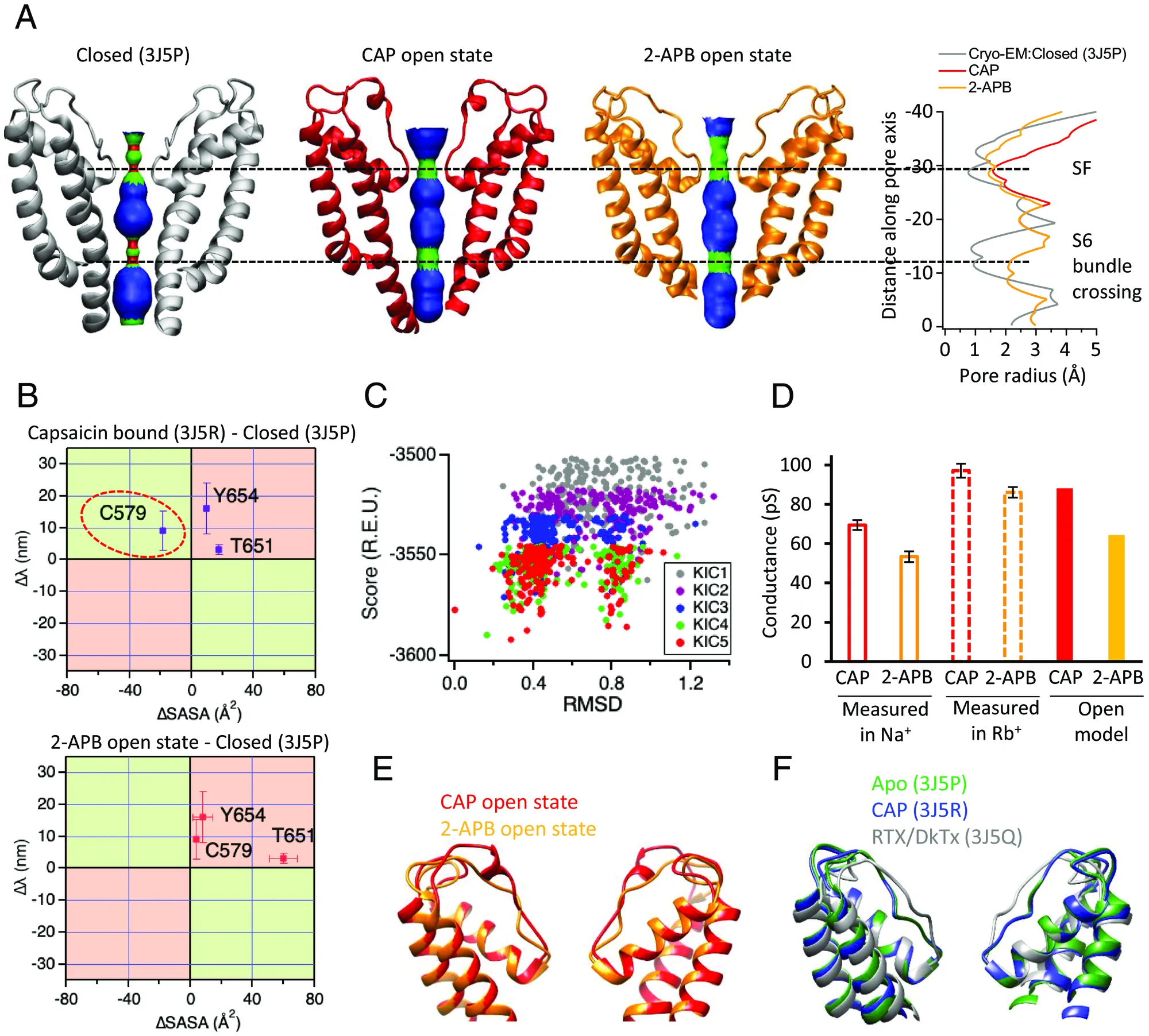
Rosetta modeling of the open states pore. (*A*) Comparison of the closed TRPV1 pore cryo-EM structure (1st panel), the capsaicin-activated (2nd panel) and 2-APB -activated (3rd panel) pore models. The aqueous pore is predicted using the HOLE program. *Right* panel, comparison of the pore diameters along the vertical axis. (*B*) Scatter plot of the ANAP spectral shifts and the correlated changes in SASA for (*Top* panel) capsaicin-bound TRPV1 cryo-EM structure, and (*Bottom* panel) the 2-APB-activated open state models. (*C*) Improvements of the Rosetta energy score over five rounds of KIC modeling. (*D*) Comparison of predicted pore conductance levels in Na^+^ and Rb^+^ solutions to experimentally determined values. (*E*) Overlay of the capsaicin (red) and 2-APB (gold) activated pore models. (*F*) An overlay of the original TRPV1 cryo-EM structures solved in the apo (PDB entry 3J5P, green), capsaicin-bound (3J5R, blue), and RTX/DkTx-bound (3J5Q, silver) states is shown for comparison. Error bars indicate mean ± SEM.

Currently there is no reported 2-APB-induced open state of TRPV1 solved by cryo-EM. To visualize possible 2-APB-induced conformational changes, we turned to computational modeling. Our fluorescence measurements revealed that channel residues at different locations undergo distinct degrees of exposure during channel activation. We have previously shown that this information can serve as a powerful guide for computational modeling of channel conformation using the Rosetta suite (26). Using the degrees of ANAP exposure to constrain the increase in solvent accessible surface area (SASA) at multiple channel sites, we computed and compared structural models of the activated states induced by capsaicin and 2-APB (Fig. 4 *A*–*C*). The predicted 2-APB open state model differs from the capsaicin open state model we previously reported (26) and the RTX open states solved by cryo-EM (20, 24, 38) (*SI Appendix*, Figs. 11 and 12). To assess reliability of the computational models, the ion conductance of each model was predicted by the HOLE program (39). The predicted conductance levels of these models matched well to our experimental observations (Fig. 4*D*). Consistent with results from fluorescence measurements, the 2-APB-induced open state model overlapped with the capsaicin-induced open state model around the inner gate but deviated substantially at the outer pore region (Fig. 4*E*). The difference is reminiscent of that observed between the cryo-EM structures of capsaicin-bound and RTX/DkTx-bound channels (Fig. 4*F*). In summary, structural modeling results support the presence of distinct open conformations of TRPV1 induced by distinct activators.

### Slower Gating Kinetics in the Presence of Two Agonists.

**Fig. 5.**
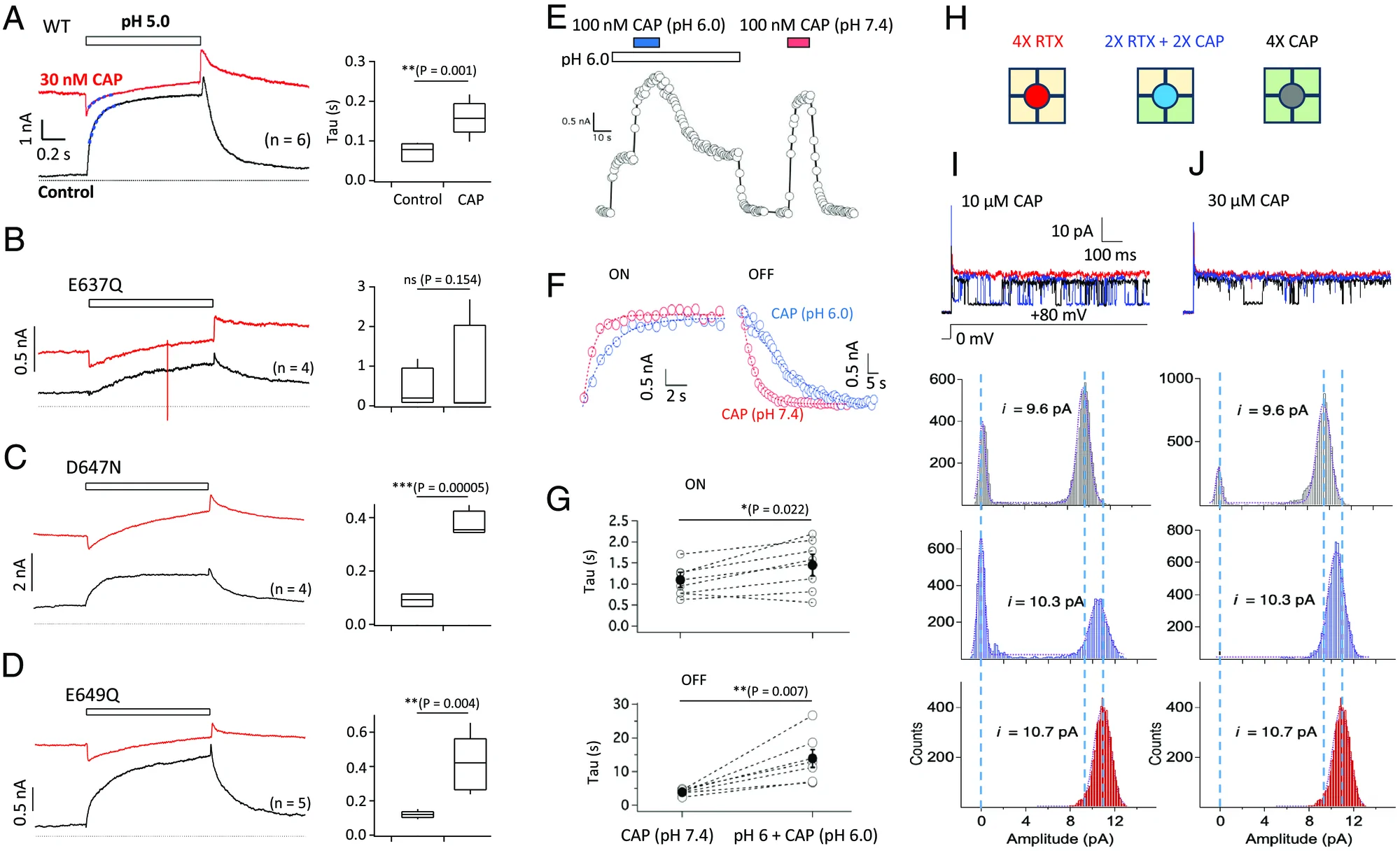
Slower rate of TRPV1 proton activation in the presence of capsaicin. (*A*–*D*) Representative whole-cell patch-clamp recordings at +60 mV of TRPV1 WT (*A*) and mutants of putative proton binding sites E637Q (*B*), D647N (*C*), and E649Q (*D*) *Left* panels, current traces from the same cell induced by protons at pH 5.0 alone (black) and in the presence of 30 nM capsaicin (red). *Right* panels, box-and-whisker plots of the current raising-phase time constants measured during pH 5.0 application. The whisker top, box top, line inside the box, box bottom, and whisker bottom represent the maximum, 75th percentile, median, 25th percentile, and minimum value of each pool of tau measurements, respectively. Superimposed in panel A are fits of a single-exponential function to the current raising (blue dotted curves), from which the time constant (Tau) was determined. n = 4 to 6. (*E*) A representative outside-out patch recording for which protons were applied before capsaicin. (*F*) Comparison of current activation (ON) and deactivation (OFF) phases. Superimposed are fits of an exponential function. (*G*) Summaries of current time constants. Filled symbols represent mean values. (*H*) A cartoon illustrates subunits bound with capsaicin (light green) or RTX (light yellow), with the colors of the center circle corresponding to the colors used for current traces and histograms. (*I* and *J*) Comparisons of single-channel amplitudes of a YYAA concatemeric channel activated by capsaicin (black), RTX (red), or their combination (blue). The raw data used here have been published previously in Li and Zheng (18, *SI Appendix*, Fig. S6). Superimposed on all-point histograms are fits of a double-Gaussian function. Error bars indicate mean ± SEM. Statistical significance was determined using Student’s *t* test. *P < 0.05; **P < 0.01; ***P < 0.001; ns, not significant.

If different stimuli drive TRPV1 into distinct open conformations, what happens when two stimuli are applied together? TRPV1 is a homotetramer. Because ligand binding is stochastic, the four subunits of a channel complex may transiently be in different ligand-bound states during activation. To detect such situations, we switched to kinetic measurements of current activation. We first recorded the time course of macroscopic current induced by protons at pH 5.0, which yielded near maximal activation (2, 6) (Fig. 5*A*, black trace). With the same patch, the proton activation time course was recorded again in the presence of capsaicin at 30 nM [lower than the EC50 value of 110 nM for mouse TRPV1 at room temperature; according to the MWC model, less than 50% of the subunits were expected to contain a bound capsaicin (18)] (Fig. 5*A*, red trace). When the activation rates were compared, we found that proton activation was significantly slowed down when capsaicin was present (Fig. 5 *A*, *Right* panel). The deactivation rate also appeared to be slower in the presence of both capsaicin and protons, though the averaged data did not reach statistical significance and were therefore not analyzed further. Comparable results were obtained in channels carrying mutations at one of the putative proton-binding sites, where such mutations are expected to disrupt the proton-binding step (Fig. 5 *B*–*D*). To verify that the slowing of gating kinetics is reciprocal, we reversed the order of capsaicin and proton applications (Fig. 5 *E* and *F*). Again, we observed that in the presence of protons (at pH6.0), capsaicin (at 100 nM) activated TRPV1 more slowly than it did at neutral pH; the deactivation rate was likewise slower (Fig. 5*G*).

Given the multiple plausible explanations for the slowed gating kinetics, we turned to single-channel recordings from the concatemeric channel YYAA. In this concatemer, the Y subunits are WT to which RTX binding is irreversible, and the A subunits are Y512A mutants to which RTX binding is reversible (17, 31). We first used capsaicin (at 10 μM or 30 μM) to activate the YYAA concatemeric channel, followed by saturating RTX; after washing off RTX from the A subunits, capsaicin was applied again to bind the A subunits (Fig. 5*H*). As expected, the single-channel current amplitude was higher when the concatemeric channel was activated by RTX than by capsaicin as we observed from WT channels (Fig. 5 *I* and *J*). However, the open events induced by a mixture of capsaicin and RTX exhibited intermediate conductance compared to open events induced by capsaicin or RTX alone, suggesting that the open pore conformation is distinct from those induced by a single type of agonist. Thus, the slower activation kinetics in the presence of a mixture of activators are best explained by conformational clashes among subunits that create mutual impediments to their activation transitions.

## Discussion

As a polymodal nociceptor, TRPV1 ion channels respond to diverse physical and chemical stimuli that elicit distinct sensory profiles (2, 40, 41). These stimuli exhibit synergetic effects toward activating TRPV1, yet they affect separate channel structures that are coupled to the channel pore via nonoverlapping pathways (22). Our functional data reported here, together with the latest structural data (20), establish that TRPV1 activates into distinct open-pore conformations. Existence of distinct functional open states presents attractive opportunities to achieve analgesics by selectively inhibiting those induced by noxious stimuli while sparing the heat activated open state.

TRPV1 channel is a nociceptor whose activation produces a depolarizing current—due to nonselective permeation of cation ions—to excite sensory neurons. It is interesting to note that TRPV1 contains an ion selectivity filter sequence (TIGMGD) nearly identical to that of potassium channels (TIGYGD), which are highly selective for K^+^ and produce hyperpolarizing currents. Structurally, the Kv channel selectivity filter conformation is secured by a characteristic tyrosine-mediated hydrophobic scaffold supported by the pore helices (42), a feature not observed in the TRPV1 cryo-EM structures (43). The Kv selectivity filter is a long water-filled pathway lined with multiple binding sites optimized for binding potassium ions. The corresponding structure of TRPV1 nonetheless has a wide V-shaped entrance with only the G643 residue defining the selectivity filter and the associated poor ion selectivity (*SI Appendix*, Fig. S13). The TRPV1 pore is also thought to be highly flexible, undergoing dramatic dilation upon extended opening that alters ion selectivity as detected by the ion permeation ratio (44). Mutations to outer pore and surrounding residues as well as N-glycosylation at the pore turret exhibited substantial impact of the dynamic process of ionic selectivity (45, 46). Recordings in the present study were generally shorter than what it would take to detect signs of time-dependent change in conductance. Hence, the multiple open states we detected are distinct from the pore dilated state(s). Instead, they most likely reflect the multiple pore conformations observed in a recent detailed investigation of the TRPV1 structures (20). Data from that study revealed the distance between two G643 carbonyls across the pore varies between 4.4 Å in the apo state to 6.7 Å in a fully open state, with multiple intermediate distances for other identified states, some of which are thought to be conducting states. If these open pore conformations are present under physiological conditions, they are expected to yield distinct ion selectivity profiles as we detected in the present study through single-channel conductance and AFME measurements.

In conclusion, our present study identified distinct functional open states of TRPV1 that may correspond to the open pore structures revealed by cryo-EM. One may wonder whether the different single-channel conductance levels we observed can arise from the well-known filtering effect of patch-clamp recording on rapid transitions between the closed and open states (29). Subconductance states observed in *Shaker* potassium channels, for example, may result from such effects (47, 48). Applying this interpretation to the different single-channel conductance levels observed in TRPV1 is admittedly attractive, as it does not violate the MWC model that successfully describes TRPV1 activation by vanilloids (17–19). Indeed, when AMFE measurements revealed changing ion permeation properties of TRPV1, we originally interpreted these findings as reflecting activation into a flickering open state whose stability is determined by individual activators (49). However, the identification of multiple stable pore conformations in a recent cryo-EM study (20) suggests that, although TRPV1 may operate according to the MWC principle in the presence of a single type of activator, different activators may stabilize distinct open states. The observation that capsaicin- and RTX-activated opening events differ drastically in conductance yet exhibit similar noise levels (*SI Appendix*, Fig. S2) is consistent with this interpretation. It is also known that when TRPV1 is completely desensitized to one stimulus, the channel can still respond to other stimuli (7, 50). Importantly, even if mechanistically the multiple conductance states of TRPV1 originate from a single open conformation whose stability varies depending on the activator, our present study demonstrated that these states are functionally distinct under near-physiological conditions—a distinction that can be exploited pharmacologically for analgesic purposes.

Besides guiding practical approaches to regulate TRPV1 activities, findings from the present study offer clues for understanding biological polymodal receptors. It is generally held true that evolution produces low-energy protein conformations that yield discrete functional states selected by specific biological niches. The number of states in well-studied proteins are small: It is not uncommon that a protein exists in just two states, a resting (or relaxed) state and an activated (or tense) state. Formation of multisubunit complexes has the potential of increasing state numbers though it is not a default: As the MWC model elegantly illustrates, a homomultimer can operate as a two-state system while exhibiting markedly enhanced sensitivity to agonist through cooperative interactions. How proteins respond to molecules that have not served as evolutionary drivers remains poorly understood. This question becomes more pressing as AI-aided drug developments produce protein binders, many targeting sites with no known natural ligands, at an accelerating pace. In the case of TRPV1, we previously found that the MWC model applied nearly perfectly to the energetics of capsaicin and RTX activation (17–19). The present study revealed that these two competitive agonists yield different conductance states. Similar phenomena had been reported in the literature. For example, lysophosphatidic acid yields higher conductance than capsaicin (28), so does the centipede peptide toxin RhTx at negative membrane potentials (51). The spider toxin DkTx on the other hand produces lower conductance (4, 52). The present study revealed different structural dynamics induced by distinct TRPV1 stimuli that support a uniform framework to explain these observations.

## Methods

### Molecular Biology and Cell Transfection.

Murine and rat TRPV1 cDNA were used in this study. An eYFP cDNA was fused to the C terminus of the channel cDNA to help identify channel-expressing cells. The eYFP tag did not exhibit detectable effect on functional properties of TRPV1 (35). Mutant and concatemeric channel constructs used in this study have been previously reported (17). pANAP vector was purchased from Addgene.

HEK293T cells were purchased from American Type Culture Collection (ATCC) and authenticated to be contamination-free. Cells were cultured in a Dulbecco’s modified eagle medium with 10% fetal bovine serum and 20 mM L-glutamine at 37 °C with 5% CO_2_. Transient transfection was carried out with Lipofectamine 2000 (Life technologies) according to the manufacturer’s protocol. One or two days after transfection, functional or fluorescence recordings were performed.

### Chemicals.

L-ANAP methyl ester was purchased from AsisChem. 6′-iRTX was purchased from Alomone Labs. All other chemicals were purchased from Sigma-Aldrich unless otherwise stated.

### Site-Specific Fluorescence Recording Using Unnatural Amino Acid.

Incorporation of ANAP followed the protocol used in our previous study (26). Briefly, ANAP was incorporated into TRPV1 with a TAG amber stop codon mutation (36). After cotransfection of both TRPV1 and pANAP vectors, ANAP was directly added to the culture medium to the final concentration of 20 µM. After 1 to 2 d, ANAP-containing culture medium was completely changed. Cells were further cultured in ANAP-free medium overnight before experiments. Site-specific fluorescence spectral measurement protocol was previously described (26, 35). Briefly, fluorescence recordings were made with our home-built system based on an inverted fluorescence microscope (Nikon TE2000-U). ANAP was excited by the wLS LED light source (QImaging) with a 375/28 excitation filter, T400lp dichroic mirror, and 435LP emission filter using a 40X oil-immersion objective (NA 1.3). The emission of ANAP was collected and passed through an Acton SpectraPro 2150i spectrograph; the emission spectrum was imaged with an Evolve 512 EMCCD camera. Identification of the peak emission wavelength was done by fitting the spectrum to a skewed Gauss function.

### Electrophysiology.

Patch-clamp recordings were performed with a HEKA EPC10 amplifier controlled by PatchMaster software (HEKA). Patch pipettes were pulled from borosilicate capillary using a Sutter Instrument P-97 micropipette puller and fire-polished to resistance of ~4 MΩ. For whole-cell recording, serial resistance was compensated by 60%. For single-channel recordings, patch pipettes were fire-polished to a resistance range of 6-to-10 MΩ. Both bath and pipette solutions contained the following ingredients unless mentioned otherwise: 130 mM NaCl, 10 mM glucose, 0.2 mM EDTA, and 3 mM Hepes (pH 7.2). Membrane potential was clamped at +80 mV for single-channel recordings. Current was sampled at 10 kHz and filtered at 2.9 kHz. Additional digital filtering was applied during data analysis and preparation of representative traces. All recordings were performed at room temperature. Perfusion of solutions was achieved by a gravity-driven system (RSC-200, Bio-Logic), for which different solutions were delivered through separate tubes without mixing. For measuring the activation rate, a rapid solution switching method based on theta (Θ) glass pipettes was used, as described previously (47). Two theta (Θ) glass pipettes were used during the patch-clamp recording. Switching between pipettes was done manually.

### Computational Modeling.

The modeling procedure has been described in detail in our previous publication (26). Briefly, membrane-symmetry-loop modeling was performed using the Rosetta molecular modeling suite (53) version 2015.25 to model ligand-induced open states of TRPV1. The cryo-EM structure of capsaicin bound state (3J5R) was chosen as the starting template; the selectivity filter, the pre S6 linker and the S1 to S2 linker were modeled de novo with the kinematic (KIC) loop modeling protocol (54, 55). SASA of each residue in TRPV1 structures models was measured by RosettaScripts (56) within the Rosetta suite. Differences in SASA values between the apo state cryo-EM structure (3J5P) and the capsaicin bound state (3J5R) do not match with the experimentally obtained ANAP fluorescence spectral shifts, whose polarity indicate the change in exposure upon 2-APB activation (Fig. 4 *B*, *Top* panel). Therefore, for each round of KIC modeling, the generated models were evaluated by the SASA value: Models with an increase in SASA larger than 20 Å^2^ compared to the closed state were selected, and the first 20 top-ranked models with the lowest Rosetta energy were passed to follow-up modeling. After five rounds of KIC modeling, we observed two clusters of models (Fig. 4*C*). Close inspection indicated that one cluster represented a closed state, which was excluded from further consideration; the other cluster exhibited a pore diameter similar to that of the reported cryo-EM open states (20, 24, 38). Top models of this cluster also yielded a good match between SASA changes and ANAP spectral shifts (Fig. 4 *B*, *Bottom* panel); the top model from this cluster was refined by the relax application (57) within the Rosetta suite. FSC between the model and the capsaicin bound state electron density map (EMD ID: 5777) was calculated by Rosetta.

Pore radius of a TRPV1 model was calculated by the HOLE program version 2.0 (39). All the molecular graphics of TRPV1 models were rendered by UCSF Chimera (58) software version 1.12.

### Statistics.

All experiments were independently repeated at least three times. Data are presented as mean ± SEM. All statistical analyses were performed using GraphPad Prism. For comparisons among multiple groups, ordinary or repeated-measures one-way ANOVA followed by Tukey’s multiple comparisons tests was used, as specified in the corresponding figure legends. Student’s *t* test was used for comparisons between two groups, with paired tests applied according to the experimental design. “ns” indicates no significance. *P < 0.05; **P < 0.01; ***P < 0.001.

## Supplementary Material

Appendix 01 (PDF)

## Data Availability

Study data are included in the article and/or *SI Appendix*.
