# Supplementary material for: Distinct functional open states of the polymodal nociceptor TRPV1
Source: Proc Natl Acad Sci U S A. 2026 Oct 15;123(42):e2614335123. doi: 10.1073/pnas.2614335123 (PMC13684282; doi:10.1073/pnas.2614335123)
Supplement: Supplementary file 1 [file pnas.2614335123.sapp.pdf]

Supporting Information for

Distinct Functional Open States of the Polymodal Nociceptor TRPV1

Bo Hyun Lee[1,2], Shisheng Li[1,#], Simon Vu[1,&], Zhihao Yao[1], Yuhua Tian[1,3], Vladimir Yarov-Yarovoy[1], Jie Zheng[1,4,*]

* Corresponding author: Jie Zheng
Email: jzheng@health.ucdavis.edu

This PDF file includes:

Figures S1 to S13
Tables S1 to S2
SI References

Figures

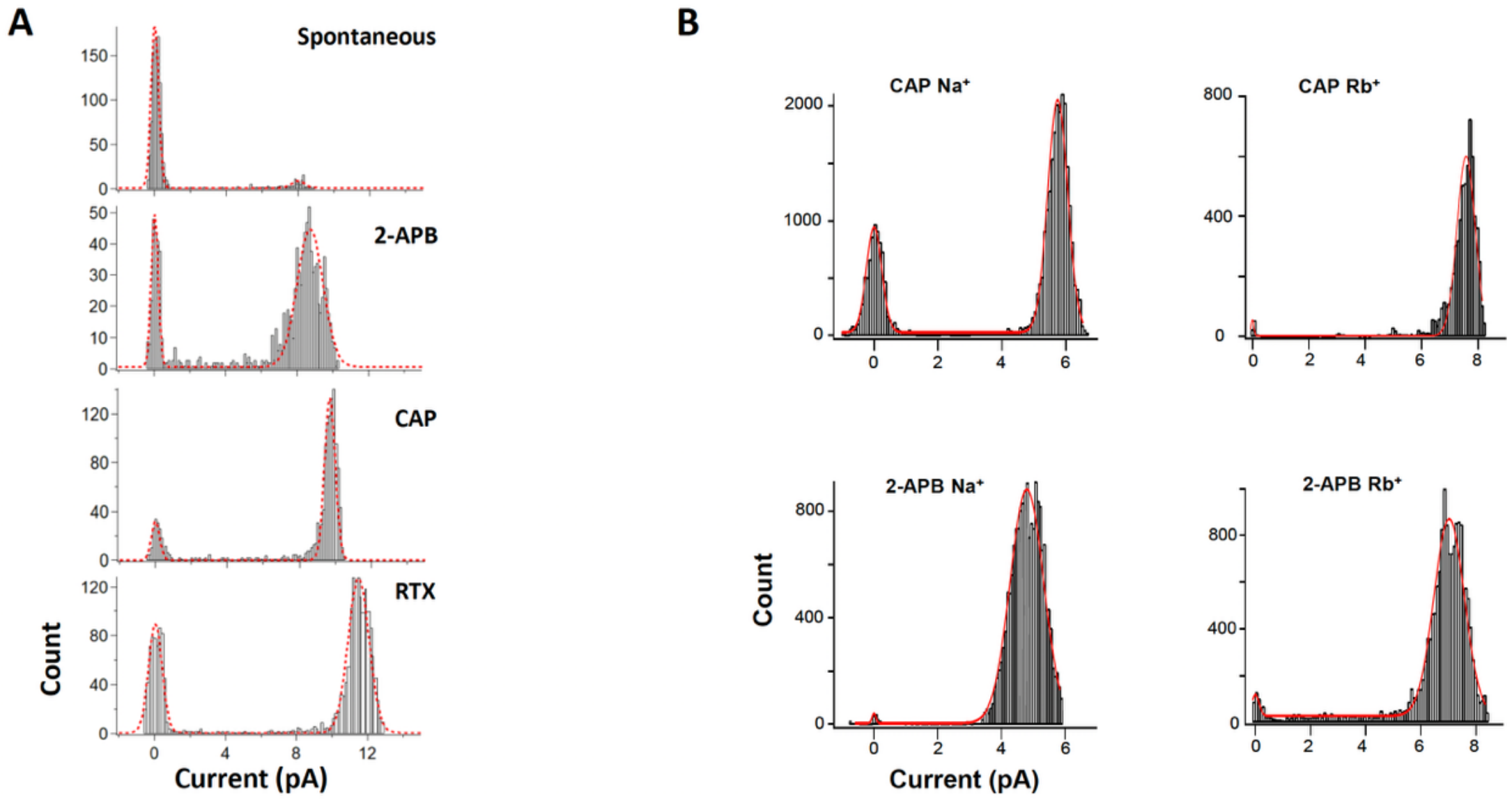

Supplementary figure S1. A. All-point histograms of currents from the same single-channel recording of TRPV1 activated by spontaneous events, 2-APB, capsaicin or RTX from the same inside-out patch-clamp recording. B. All-point histograms of currents from the same single-channel recording of TRPV1 activated by capsaicin and 2-APB recorded in Na^+ and Rb^+ solutions. All recordings were done at +80 mV. Red traces represent fits of a double-Gaussian function.

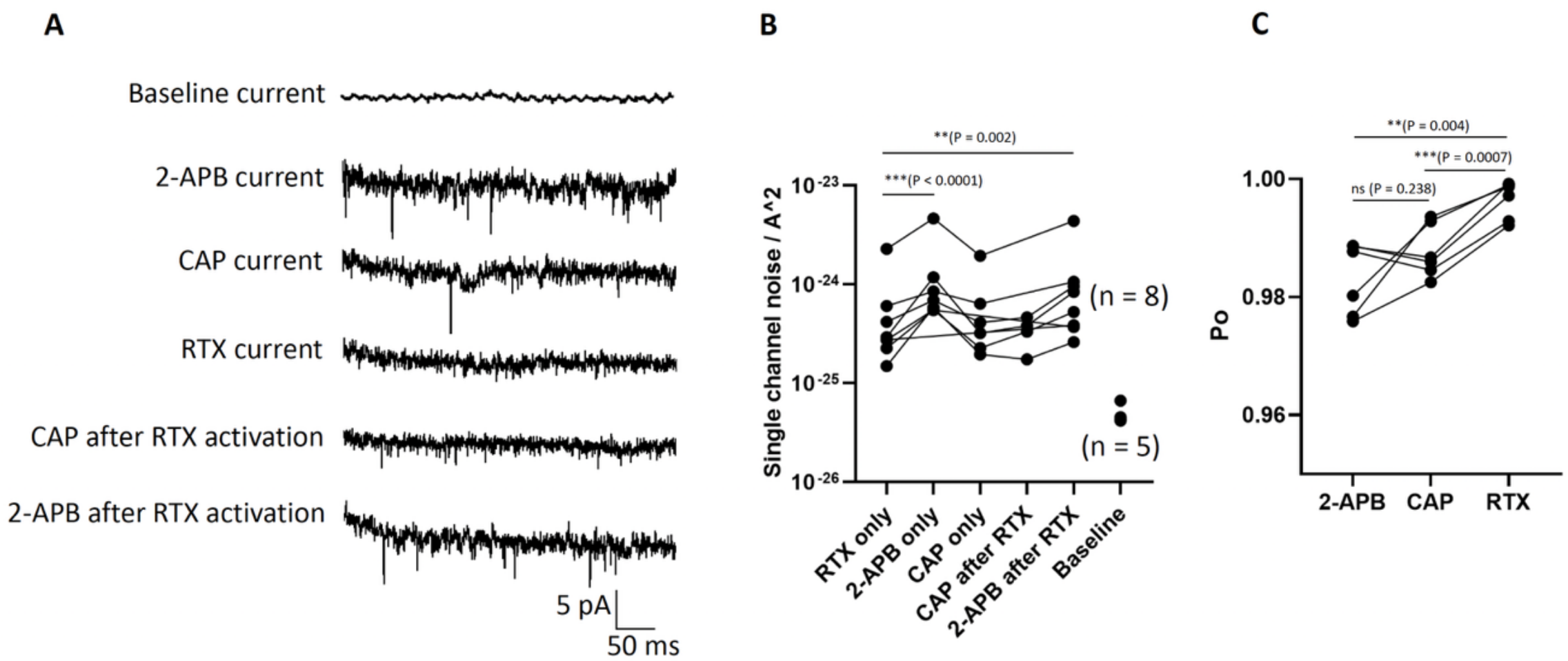

Supplementary figure S2. Open channel noise analysis. A. Representative single-channel current traces representing the open state activated by 2-APB, capsaicin, RTX or their combinations from the same inside-out patch-clamp recording at +80 mV. A trace of the closed state (baseline) is shown in comparison. Raw current traces were digitally filtered at 0.5 kHz. B. Comparison of single channel noise. C. Comparison of open probability values. n = 6. Statistical significance was determined by repeated-measures one-way ANOVA followed by Tukey's multiple-comparisons test. **, $P < 0.01$ and ***, $P < 0.001$; ns, not significant.

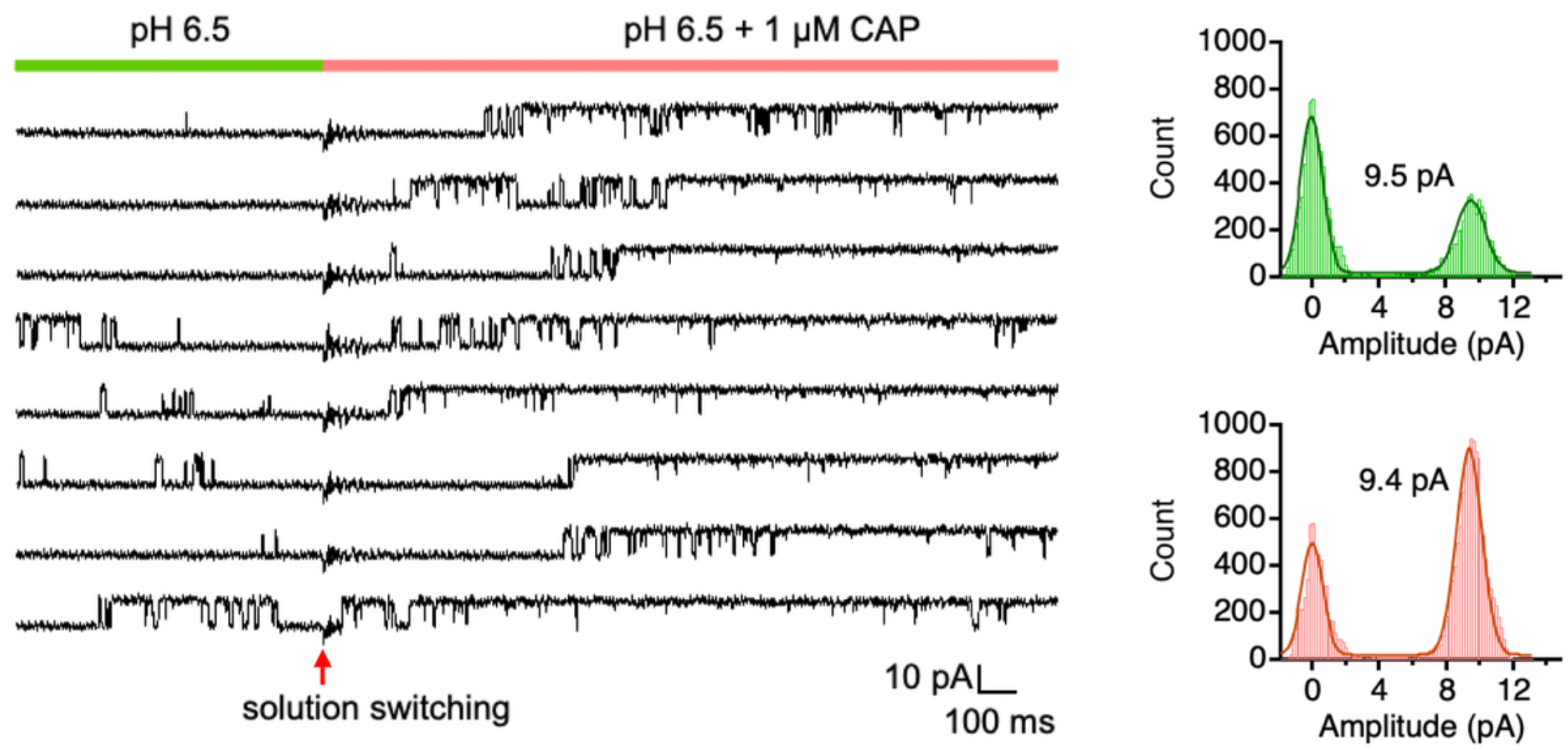

Supplementary figure S3. Comparison of single-channel amplitudes for open events induced by extracellular protons alone and those induced by capsaicin and protons. Left panel: Representative current traces from an inside-out patch recording with a pipette solution at pH6.5. At the time point indicated by a red arrow, the perfusate solution was switched from the bath solution to one containing 1 μM capsaicin using a theta glass perfusion system. Right panels: All-point histograms of single-channel events induced by protons alone (top) and those by capsaicin and protons together (bottom). Superimposed is a fit of double-Gaussian function.

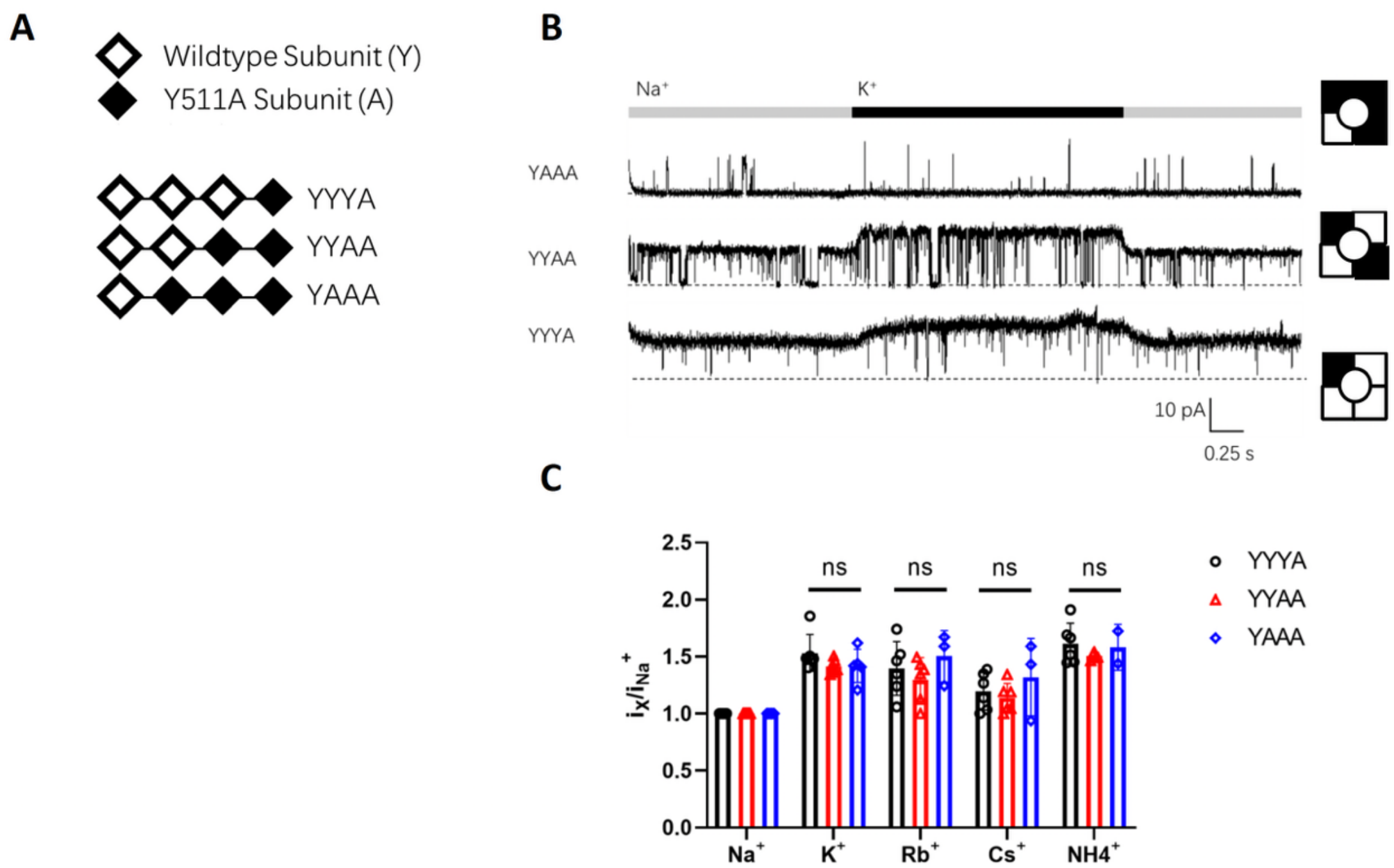

Supplementary figure S4. Ion permeation ratio measurements from concatemers with different number of bound ligand molecules. A. Diagram illustrating the design of concatemers of rat TRPV1 wildtype (Y) and Y511A (A) mutant subunits. RTX binding to the Y subunits is irreversible; binding to the A subunit units is reversible. Fully loading the concatemers with RTX followed by thorough wash yielded channels with only the Y subunits containing a bound RTX, allowing isolation of channel states with 1-to-3 bound RTX molecules. Channels with zero or 4 bound RTX molecules can be studied using wildtype channels and hence are not included here. B. Representative inside-out single-channel recordings of the concatemers with 140 mM Na^+ or K^+ in the intracellular solution. Pipette solution contained 140 mM Na^+. C. Single-channel amplitude ratios with different intracellular permeant ions (140 mM Na^+, K^+, Rb^+, Cs^+, or NH_4^+). n = 2 to 5. ns, not significant.

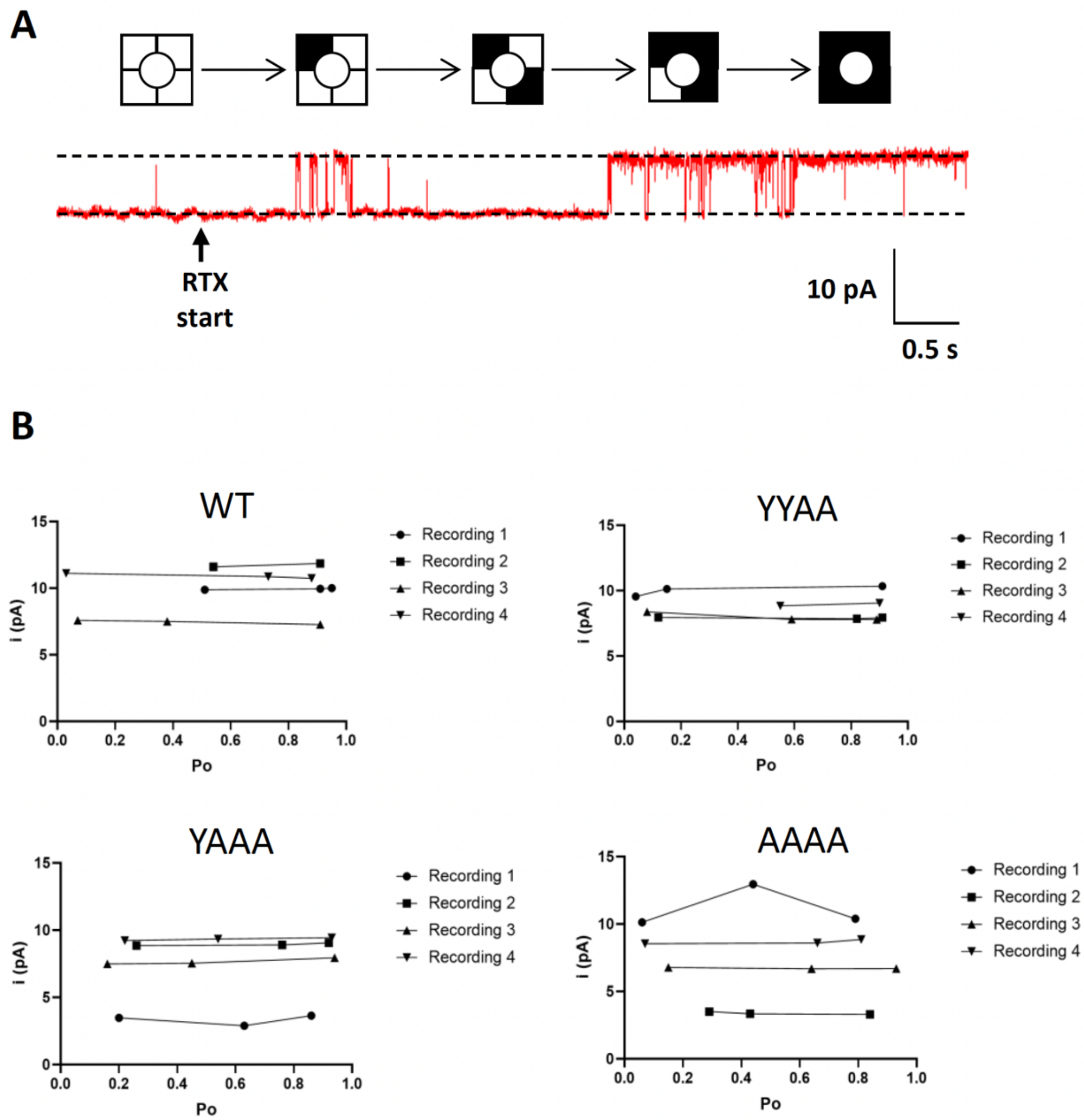

Supplementary figure S5. TRPV1 activated by varying numbers of bound ligand molecules exhibits identical single-channel current amplitude. A. A representative inside-out single-channel recording of the WT mouse TRPV1 channel during the course of RTX activation. Open probability increased as more RTX molecules bind to the channel, whereas the single-channel amplitude remained the same. B. Summaries of observations from mouse wildtype channels and rat concatemers of wildtype and Y511A subunits, confirming the same single-channel amplitude during RTX activation, except for spontaneous events in the absence of ligands.

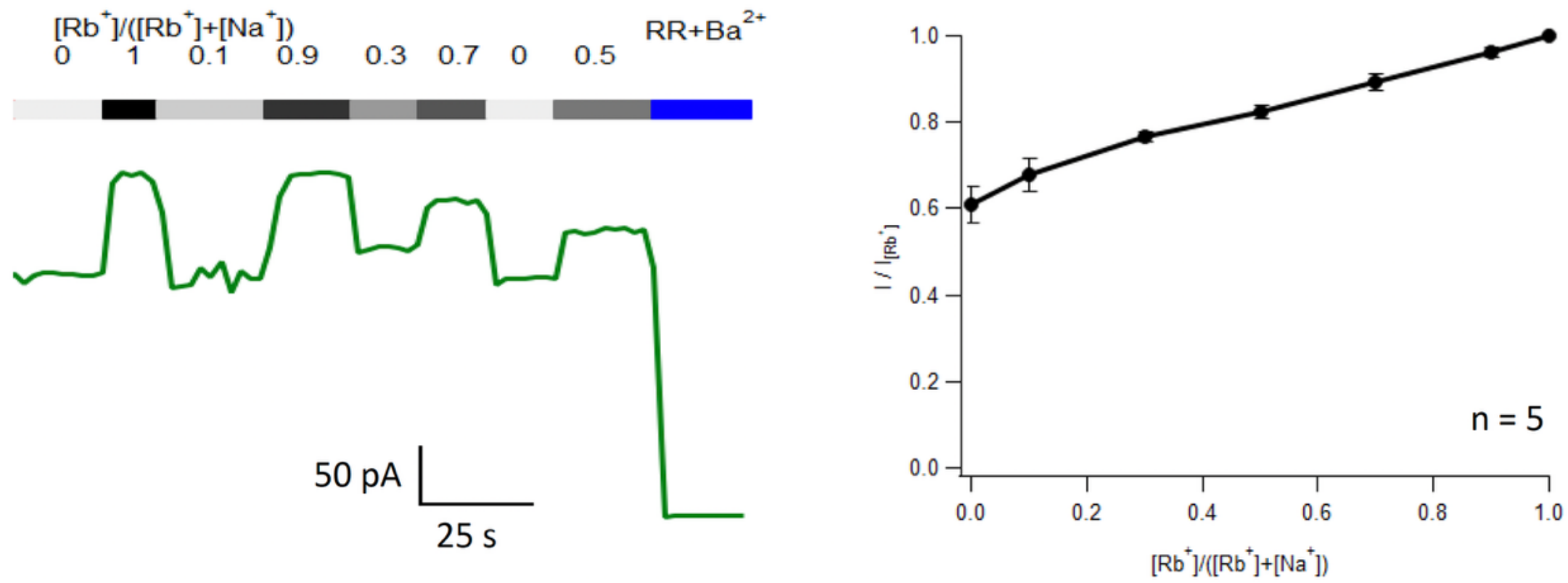

Supplementary figure S6. AMFE test of RTX activated WT mouse TRPV1 channels. Left panel: A representative macroscopic inside-out recording with differing intracellular permeate ions concentration ratios. Pipette solution contained 140 mM Na^{+}. RR, ruthenium red, a TRPV1 blocker. Right panel: Summary of AMFE test results. n = 5. Error bars indicate mean ± SEM.

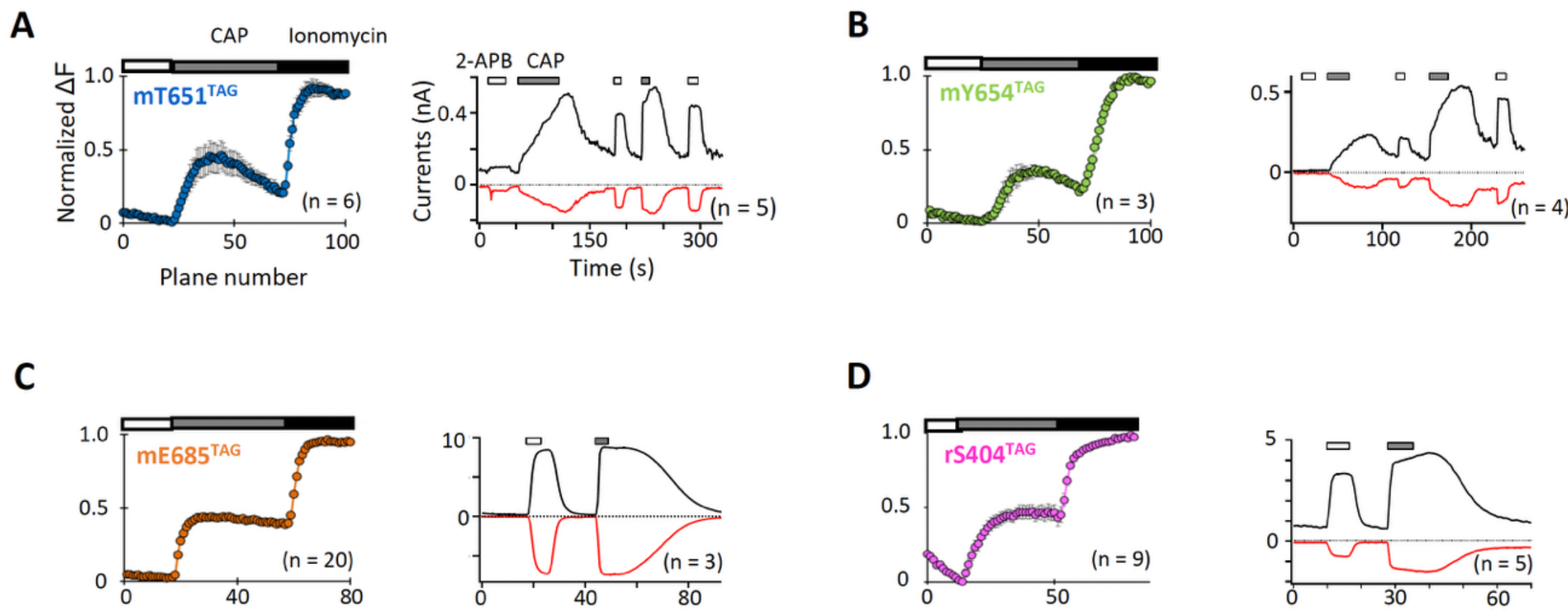

Supplementary figure S7. Functional confirmation of ANAP-labeled channels using calcium-imaging and whole-cell patch clamp recording. Left, Intensity of Ca^{2+} imaging signals (normalized to that in response to ionomycin) of mT651^{TAG} (A), mY654^{TAG} (B), mE685^{TAG} (C), rS404^{TAG} (D) transfected HEK293R cells; data in A and B have been reported in our previous publication (1) and are adapted here under the Creative Commons Attribution 4.0 International License (CC BY 4.0). 10 µM capsaicin was applied to the recording chamber, followed by 10 µM ionomycin as a positive control. Right, Representative whole-cell patch-clamp recording traces at ± 80 mV in response to both 10 µM capsaicin and 3 mM 2-APB were used to test the function of ANAP-labelled channels. n = 3-20.

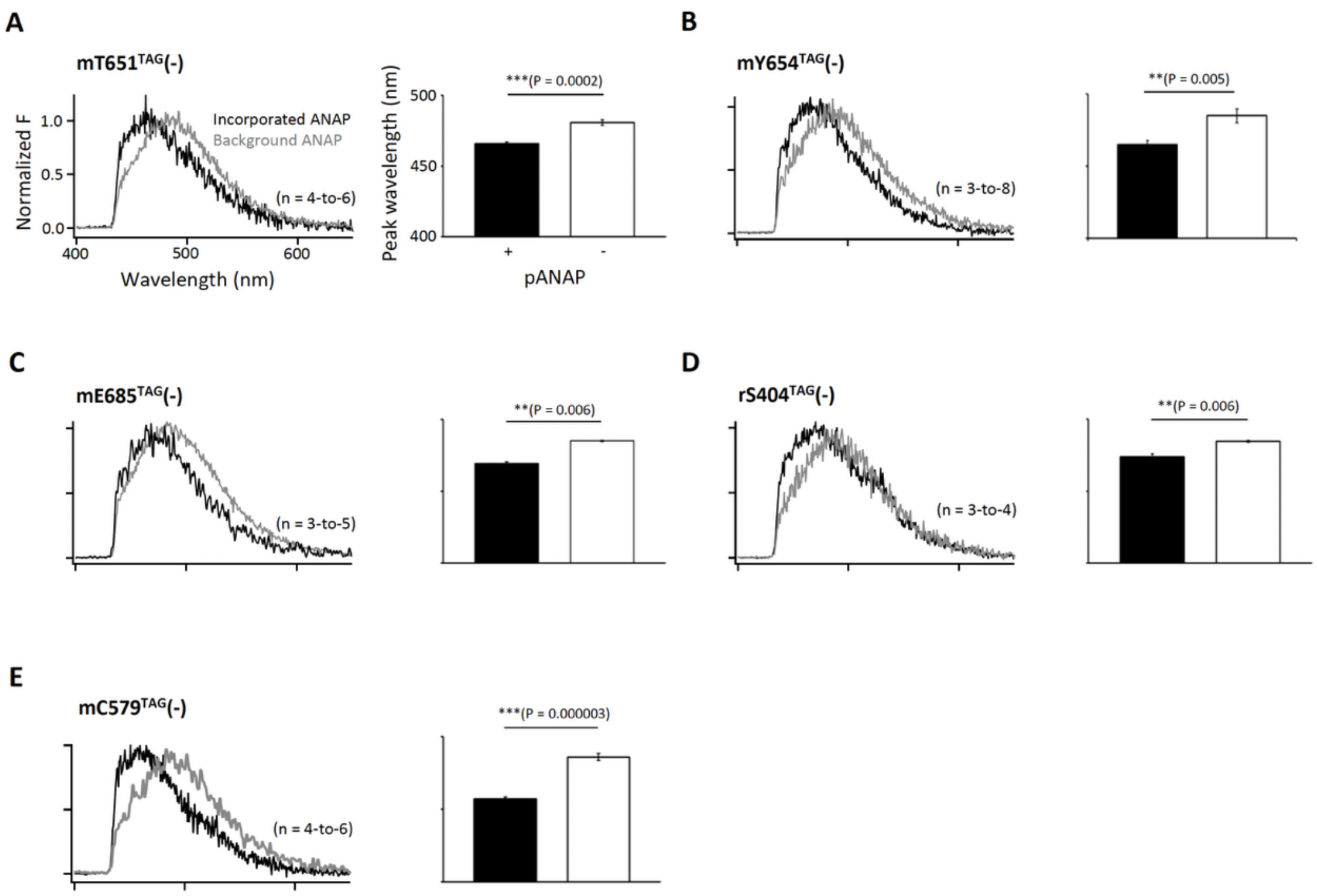

Supplementary figure S8. ANAP-incorporated channels showed different emission peaks compare to channels that lacks pANAP vector. Left, representative ANAP emission spectra of T651^{TAG} (A), Y654^{TAG} (B), E685^{TAG} (C), S404^{TAG} (D), C579^{TAG} (E) transfected HEK293T cells (black, positive control in bath solution; gray, negative control which lacked pANAP vector in the bath solution). Right, emission peaks measured at the emission spectra from the left panel. Error bars indicate mean ± SEM. Statistical significance was determined using Student's t-test. *, P < 0.05; **, P < 0.01; ***, P < 0.001.

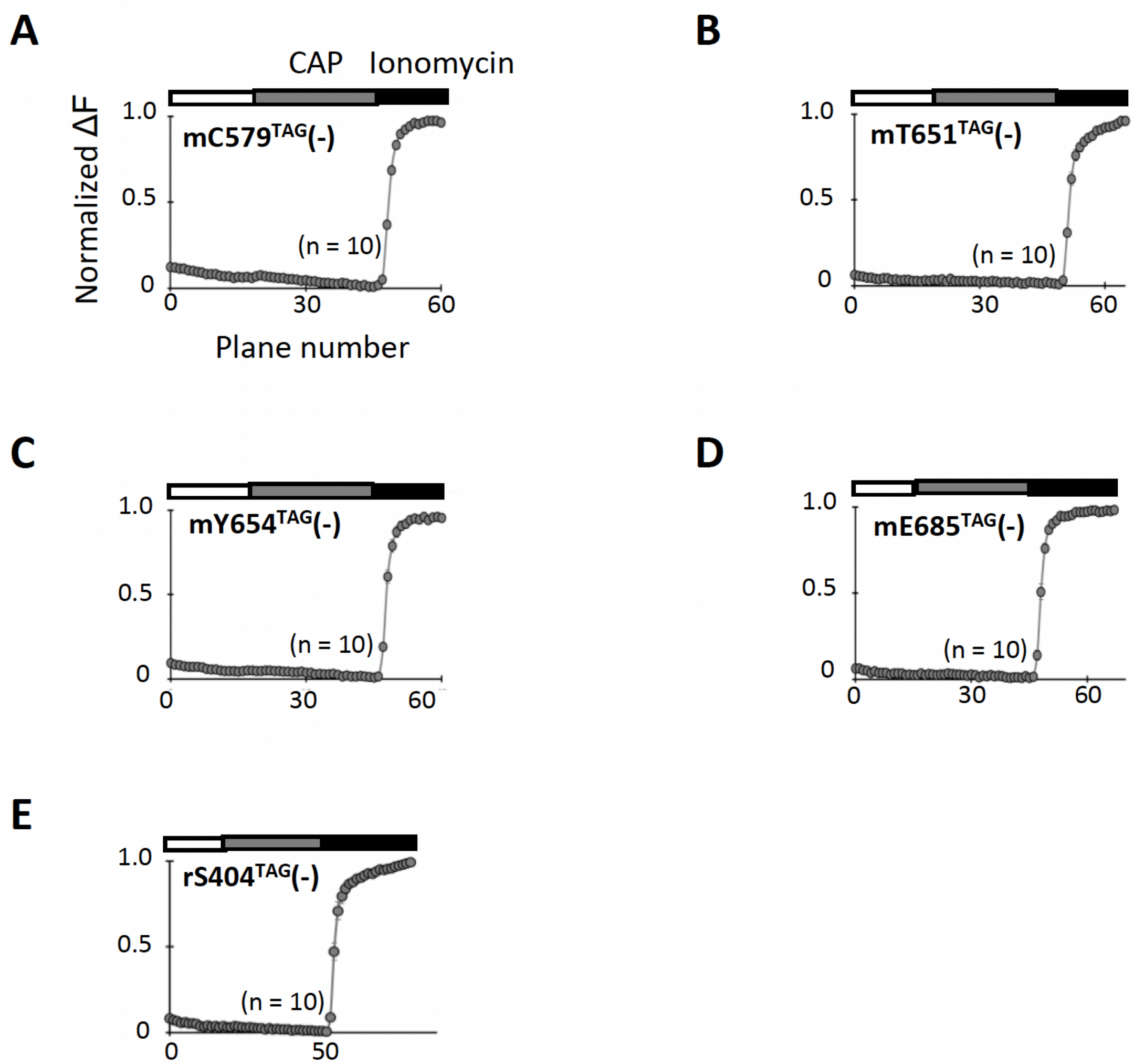

Supplementary figure S9. No functional channels were expressed in the absence of the pANAP vector. Representative Ca^{2+} imaging traces of C579^{TAG} (A), T651^{TAG} (B), Y654^{TAG} (C), E685^{TAG} (D) and S404^{TAG} (E) transfected HEK293T cells in the absence of pANAP vector. 10 µM capsaicin was applied, followed by 10 µM ionomycin as a positive control. n = 10 each.

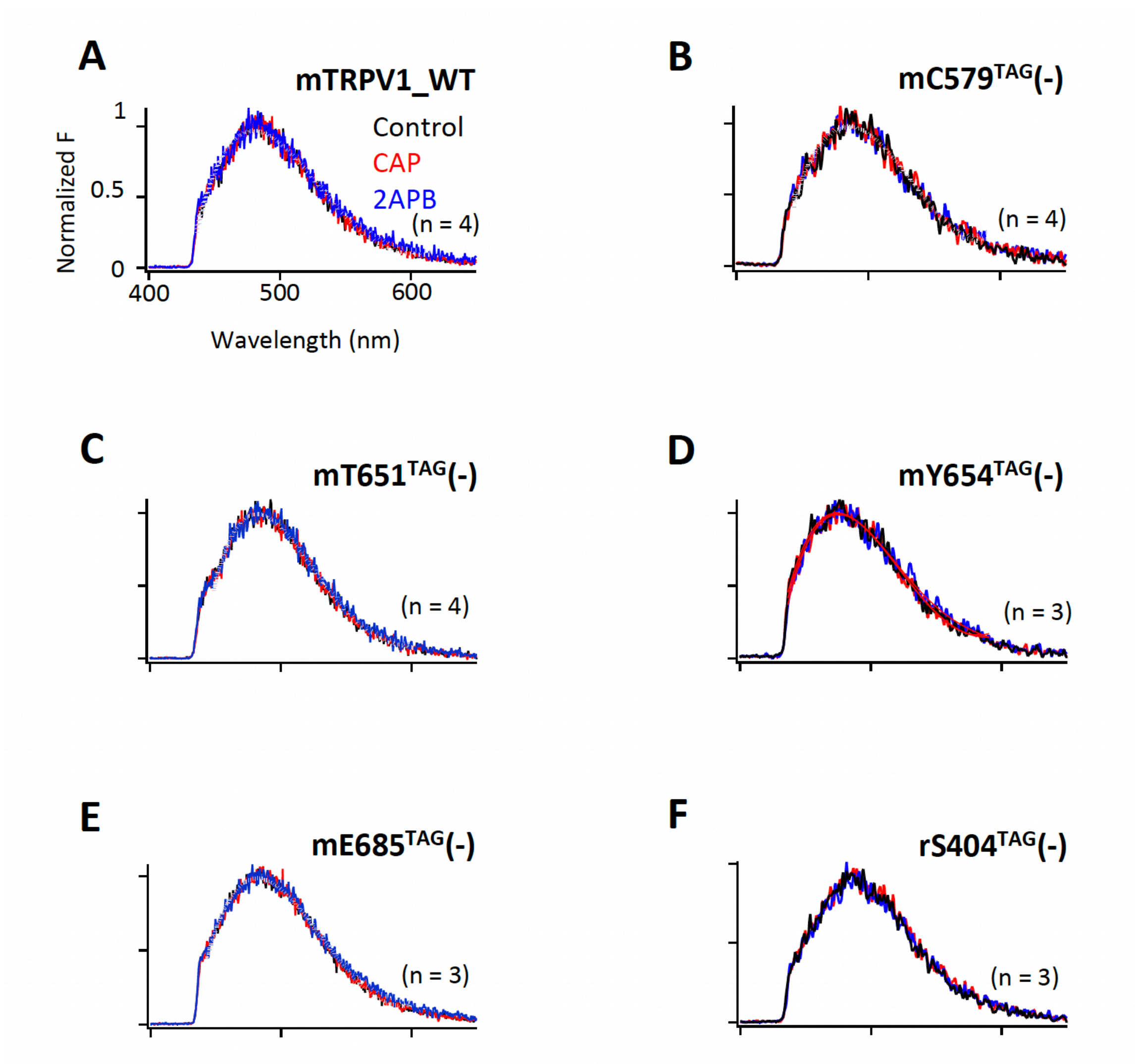

Supplementary figure S10. Cells transfected with the wildtype or TAG-encoding channel plasmids without the pANAP plasmid did not show emission peak shifts at the plasma membrane. Representative ANAP emission spectra of wild type mTRPV1 (A), $C579^{TAG}$ (B), $T651^{TAG}$ (C), $Y654^{TAG}$ (D), $E685^{TAG}$ (E) and $S404^{TAG}$ (F) transfected HEK293T cells in the absence of the pANAP plasmid. Both 10 µM capsaicin and 3 mM 2-APB were used to measure emission peaks. n = 3-4. The superimposed curves in panel D represent fits of polynomial functions.

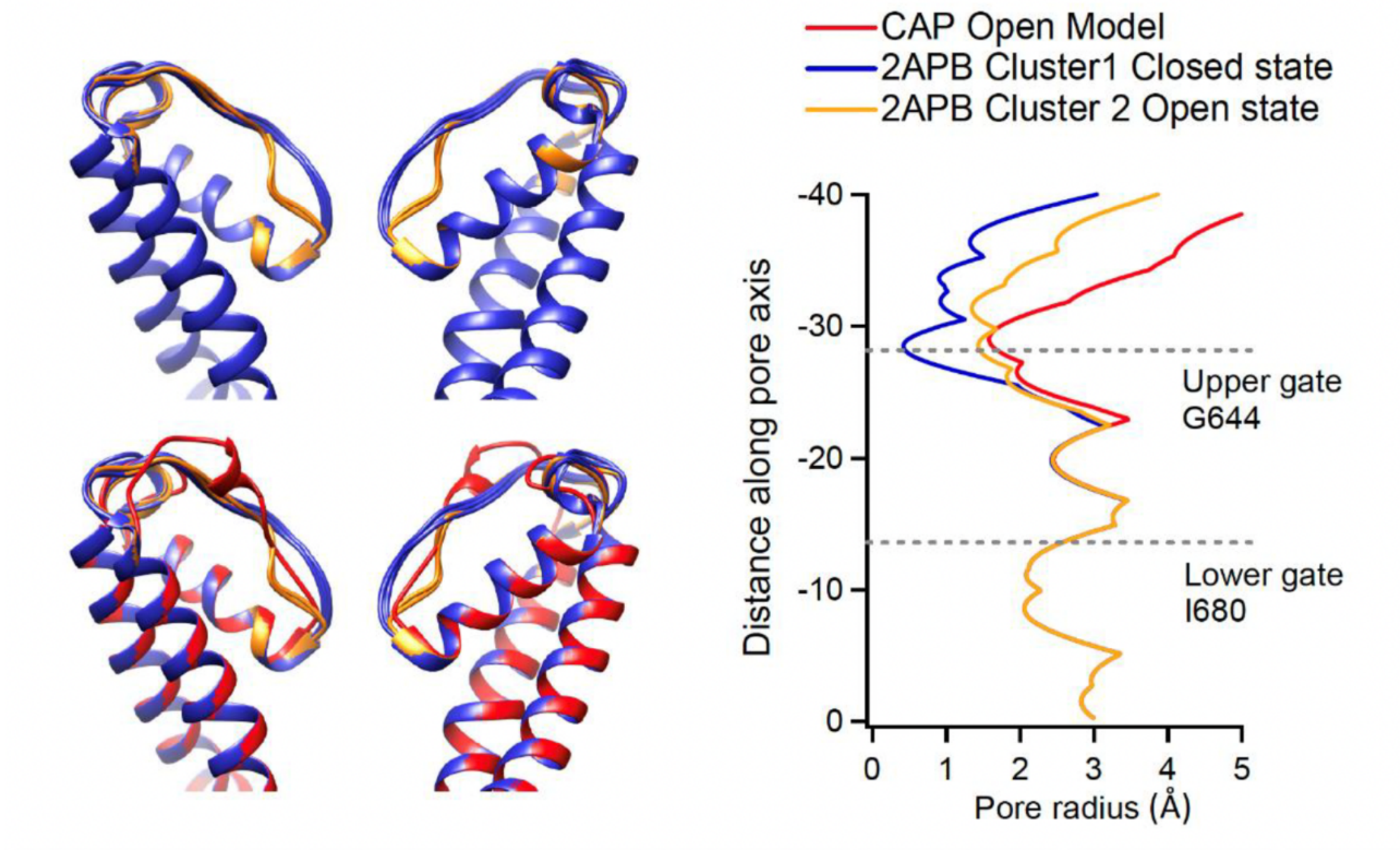

Supplementary figure S11. Left, an overlap view of the Rosetta models of TRPV1 capsaicin open state (red) and the 2-APB bound states (blue and gold). Right, comparison of the pore diameters.

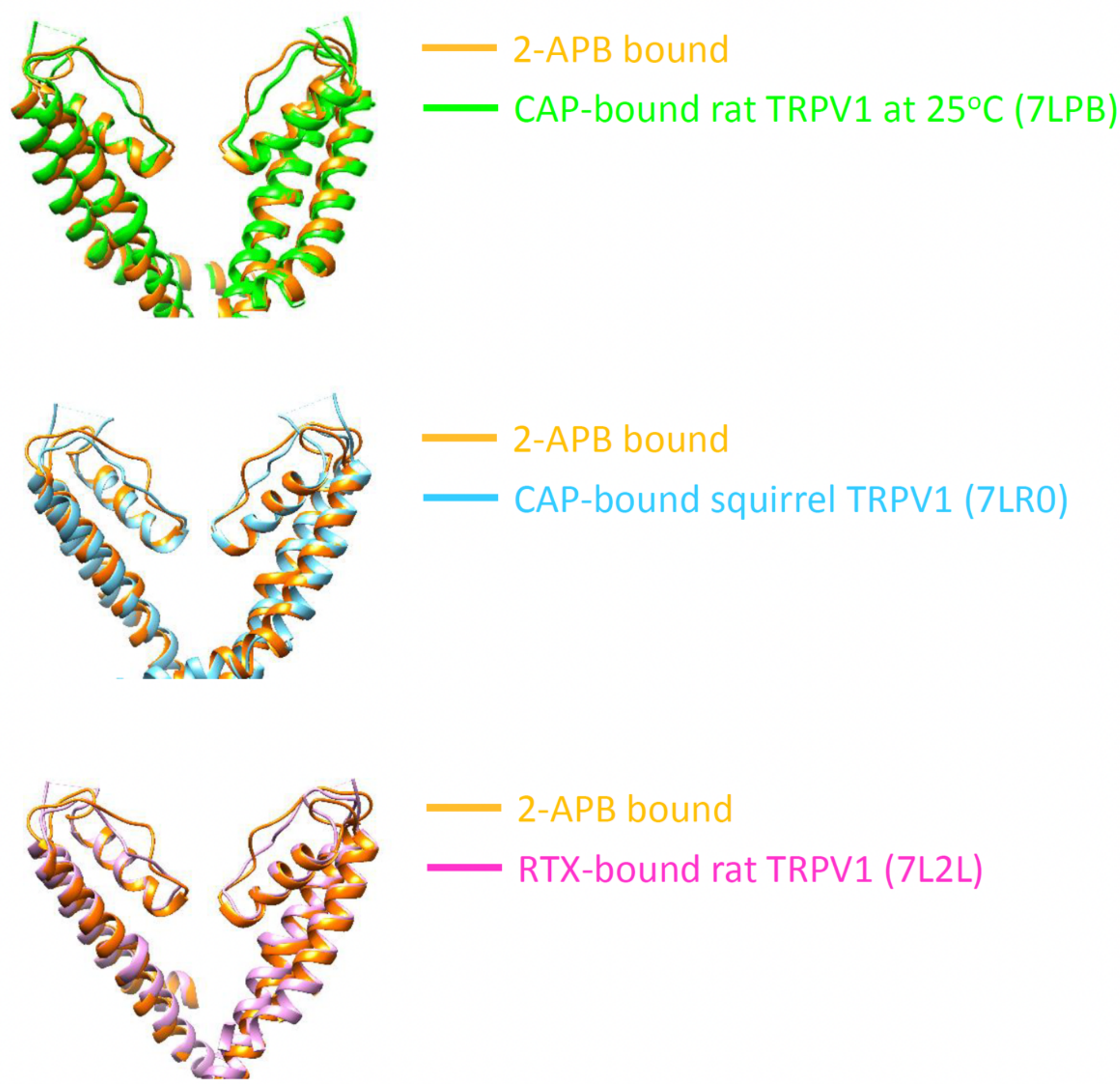

Supplementary figure S12. Comparison of 2-APB open state model with cryo-EM structures of the capsaicin bound state of rat TRPV1 at 25ºC (7LPB, top panel), the capsaicin bound state of squirrel TRPV1 (7LRO, middle panel), and the RTX bound state of rat TRPV1 (7L2L, bottom panel).

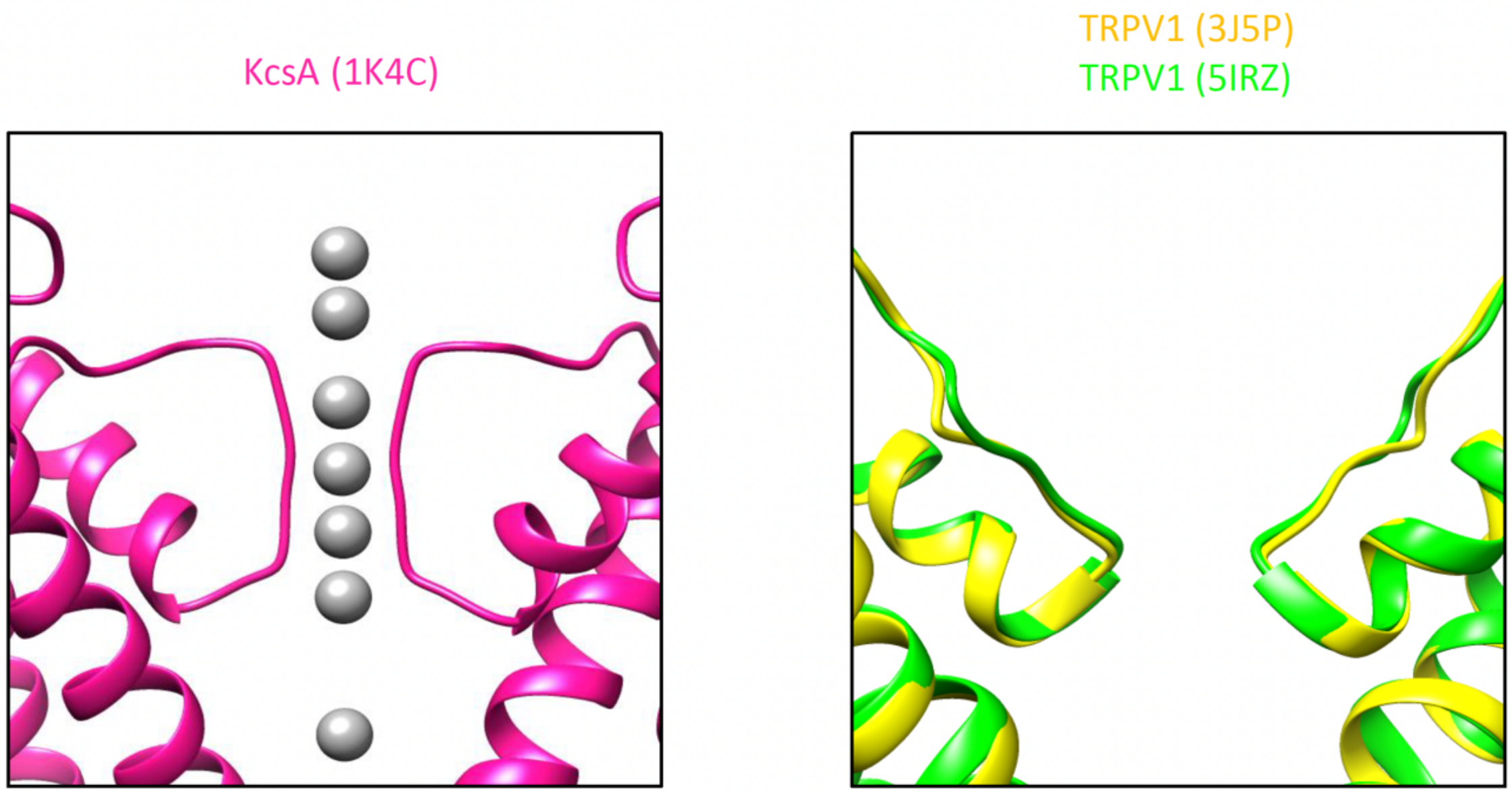

Supplementary figure S13. Comparison between KcsA and TRPV1 ion selectivity filter sequences and structures. PDB entry 1K4C, KcsA crystallography structure; 3J5P, TRPV1 cryo-EM apo structure in detergent; 5IRZ, TRPV1 cryo-EM apo structure in nanodisc. Silber balls indicate bound potassium ions. No permeant ion was identified in the shown TRPV1 structures.

Tables

Table S1. Summary of non-natural amino acid incorporation sites tested.

		Function (Ca^{2+} imaging by CAP)
S1	$F449^{TAG}$	NO
S4-S5 linker	$V568^{TAG}$	NO
	$L575^{TAG}$	N/A
	$C579^{TAG}$	YES
Turret	$I600^{TAG}$	YES
	$G603^{TAG}$	NO
	$N605^{TAG}$	N/A
Pore Helix	$S633^{TAG}$	NO
	$L636^{TAG}$	NO
	$K640^{TAG}$	NO
Pre S6	$L648^{TAG}$	YES
	$F650^{TAG}$	NO
	$T651^{TAG}$	YES
	$Y654^{TAG}$	YES
S6	$F656^{TAG}$	NO
	$Y672^{TAG}$	NO
	$I673^{TAG}$	NO
	$L676^{TAG}$	NO
	$N677^{TAG}$	NO
	$I680^{TAG}$	NO
	$G684^{TAG}$	NO
	$E685^{TAG}$	YES
	$V687^{TAG}$	NO
	$N688^{TAG}$	YES
C terminal	$F731^{TAG}$	YES
	$W750^{TAG}$	N/A
	$Y818^{TAG}$	N/A
	$F830^{TAG}$	N/A

Table S2. Summary of ANAP emission peak wavelength position measured at the plasma membrane and intracellular locations. P values represent results from Student's t-test.

	Wavelength (nm)		
	Plasma membrane	Intracellular	P value
$C579^{TAG}$	457.18 ± 1.30	466.27 ± 1.20	0.0004
$T651^{TAG}$	465.94 ± 1.29	472.96 ± 1.40	0.004
$Y654^{TAG}$	465.05 ± 2.77	472.96 ± 1.40	0.046
$E685^{TAG}$	469.31 ± 2.49	472.00 ± 2.15	0.44
$S404^{TAG}$	473.91 ± 1.90	476.22 ± 2.11	0.45

SI reference

1. F. Yang *et al.*, The conformational wave in capsaicin activation of transient receptor potential vanilloid 1 ion channel. *Nat Commun* **9**, 2879 (2018).
